# Assessing Milk Authenticity Using Protein and Peptide Biomarkers: A Decade of Progress in Species Differentiation and Fraud Detection

**DOI:** 10.3390/foods14152588

**Published:** 2025-07-23

**Authors:** Achilleas Karamoutsios, Pelagia Lekka, Chrysoula Chrysa Voidarou, Marilena Dasenaki, Nikolaos S. Thomaidis, Ioannis Skoufos, Athina Tzora

**Affiliations:** 1 Laboratory of Animal Health, Hygiene and Food Quality, School of Agriculture, University of Ioannina, 47100 Arta, Greece; a.karamoutsios@uoi.gr (A.K.); xvoidarou@uoi.gr (C.C.V.); 2 Laboratory of Food Chemistry, Department of Chemistry, National and Kapodistrian University of Athens, Panepistimiopolis Zografou, 15771 Athens, Greece; lekkap@chem.uoa.gr (P.L.); mdasenaki@chem.uoa.gr (M.D.); 3 Laboratory of Analytical Chemistry, Department of Chemistry, National and Kapodistrian University of Athens, Panepistimiopolis Zografou, 15771 Athens, Greece; ntho@chem.uoa.gr; 4 Laboratory of Animal Science, Nutrition and Biotechnology, School of Agriculture, University of Ioannina, 47100 Arta, Greece

**Keywords:** milk, species identification, proteomics, peptide biomarkers, caseins, whey proteins, analytical methods, chemometrics, authenticity

## Abstract

Milk is a nutritionally rich food and a frequent target of economically motivated adulteration, particularly through substitution with lower-cost milk types. Over the past decade, significant progress has been made in the authentication of milk using advanced proteomic and chemometric approaches, with a focus on the discovery and application of protein and peptide biomarkers for species differentiation and fraud detection. Recent innovations in both top-down and bottom-up proteomics have markedly improved the sensitivity and specificity of detecting key molecular targets, including caseins and whey proteins. Peptide-based methods are especially valuable in processed dairy products due to their thermal stability and resilience to harsh treatment, although their species specificity may be limited when sequences are conserved across related species. Robust chemometric approaches are increasingly integrated with proteomic pipelines to handle high-dimensional datasets and enhance classification performance. Multivariate techniques, such as principal component analysis (PCA) and partial least squares discriminant analysis (PLS-DA), are frequently employed to extract discriminatory features and model adulteration scenarios. Despite these advances, key challenges persist, including the lack of standardized protocols, variability in sample preparation, and the need for broader validation across breeds, geographies, and production systems. Future progress will depend on the convergence of high-resolution proteomics with multi-omics integration, structured data fusion, and machine learning frameworks, enabling scalable, specific, and robust solutions for milk authentication in increasingly complex food systems.

## 1. Introduction: Milk as a Nutritional Matrix and Target for Adulteration

Milk is a nutrient-rich fluid secreted by the female of all mammalian species, providing complete dietary sustenance for neonates [1]. Beyond its fundamental biological role, milk serves as a staple in human nutrition, consumed both in its raw form and as a key ingredient in a wide variety of dairy products, including cheese, butter, milk powder, whey powder, and fermented milk beverages [2]. Chemically, milk is a complex emulsion composed of proteins, lactose, fat globules, minerals, and numerous minor constituents, all of which contribute to its nutritional profile.

**Figure 1:**
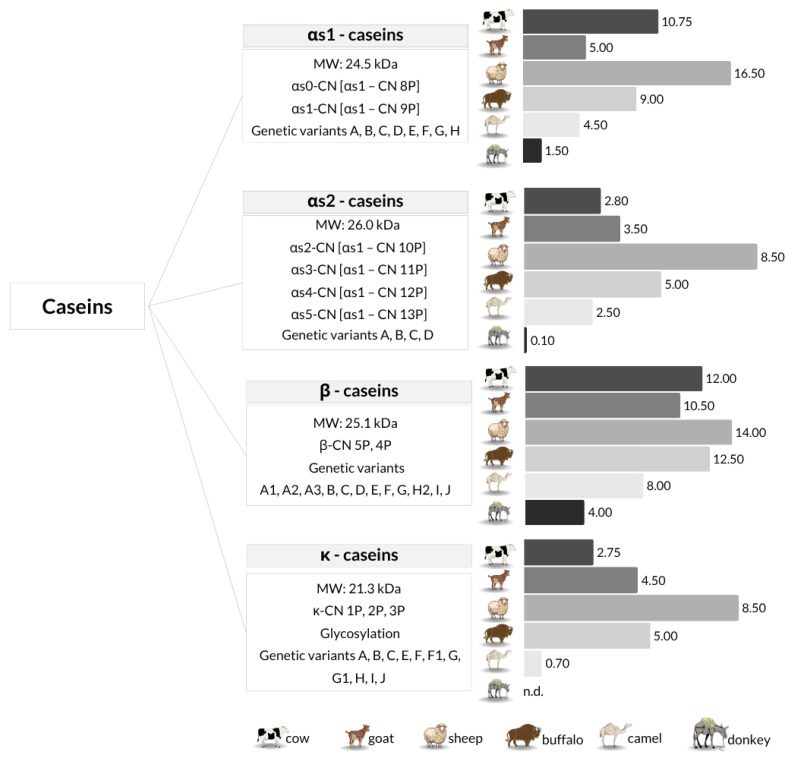
Concentrations of major casein proteins in milk from various animal species and associated molecular characteristics. Bar plots represent the average concentrations (g L^−1^) of αs1-, αs2-, β-, and κ-caseins in milk from cow, goat, sheep, buffalo, camel, and donkey, based on data from Goulding et al. (2020), Stobiecka et al. (2022), and Warakaulle et al. (2024) [4,5,6]. Annotations specify the predominant isoforms (e.g., αs1-CN) and indicate the degree of phosphorylation (P). Common genetic variants (e.g., A–H) are included to reflect species-specific polymorphisms that affect protein expression and function. The molecular weights (kDa) shown refer to canonical, unprocessed bovine sequences from UniProt: αs1-casein (24.5 kDa, P02662), αs2-casein (26.0 kDa, P02663), β-casein (25.1 kDa, P02666), and κ-casein (21.3 kDa, P02668). Animal icons represent each milk species for visual clarity.

**Figure 2:**
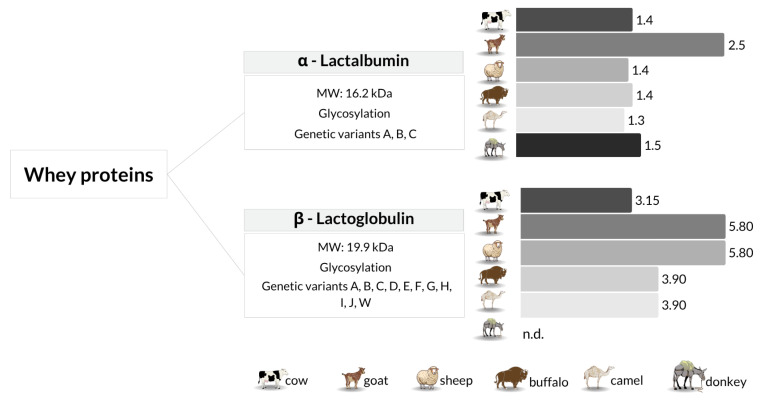
Concentrations of major whey proteins in milk from various animal species and their molecular features. Bar plots display the average concentrations (g L^−1^) of α-lactalbumin and β-lactoglobulin in milk from cow, goat, sheep, buffalo, camel, and donkey, as reported by Goulding et al. (2020), Stobiecka et al. (2022), and Warakaulle et al. (2024) [4,5,6]. Annotations indicate the presence of glycosylation summarize known genetic variants for each protein. Molecular weights (MW, in kDa) refer to canonical, unprocessed bovine sequences retrieved from UniProt: α-lactalbumin (16.2 kDa, P00711) and β-lactoglobulin (19.9 kDa, P02754).

Milk proteins are mainly divided into two main groups based on their solubility at pH 4.6, which closely matches the isoelectric point of caseins in bovine milk. However, slight variations in pH have been observed among different mammalian species, reflecting differences in amino acid composition and post-translational modifications [3]. Caseins accounts for the largest fraction of milk protein and are essential to micelle formation and mineral transport, while whey proteins contribute to immune function and biological activity. Their concentrations vary by species, reflecting differences in physiological roles and dietary needs [4,5,6]. Figure 1 and Figure 2 summarize average concentrations of these proteins across selected species.

The composition of milk and protein content varies in different animal species and also across an animal’s lifetime due to factors such as age, stage of lactation, diet, environmental conditions, and health status [7,8]. The qualitative and quantitative variability of the milk protein content as well as its effects on yield and other technological aspects can also be explained by genetic, environmental, and cattle management factors [1,9,10,11].

Milk and dairy products are highly nutritious foods widely consumed by the general population. According to a 2024–2033 report by the Organization for Economic Co-operation and Development (OECD) and the FAO of the United Nations, global per capita consumption of fresh dairy products is projected to grow by 1.0% annually over the next decade, driven primarily by rising per-capita income levels [12]. However, the increasing demand and high economic value of milk and dairy products have made the industry particularly vulnerable to fraudulent practices, raising significant concerns among consumers and authorities [13].

One common fraudulent practice in the dairy sector is the adulteration of high-value milk with lower-value milk. The primary types of milk include cow, goat, sheep, buffalo, camel, horse, donkey, and yak milk [4,5]. This practice not only undermines product quality and financial equity but also poses health risks to individuals with allergies or sensitivities. Milk proteins, especially caseins and β-lactoglobulin, often present similar structural compounds among species, leading to cross-reactivity in allergic individuals. This cross-reactivity is clinically significant, as proteins from cow, goat, and sheep milk can trigger similar allergic responses due to conserved epitopes. Thus, accurate species identification in milk contributes positively to consumer health and safety [14]. Consequently, the implementation of robust analytical methods to detect food adulteration, falsification, intentional substitution, and mislabeling is crucial across industrial, scientific, legal, and healthcare domains [15,16,17].

In addition to proteins, milk contains lipids, lactose, vitamins, minerals, and numerous bioactive metabolites that vary significantly among species. Metabolomics studies have successfully identified species-specific lipid and carbohydrate metabolic profiles, and such metabolites have been revealed as biomarkers valuable in milk authentication [18].

**Figure 3:**
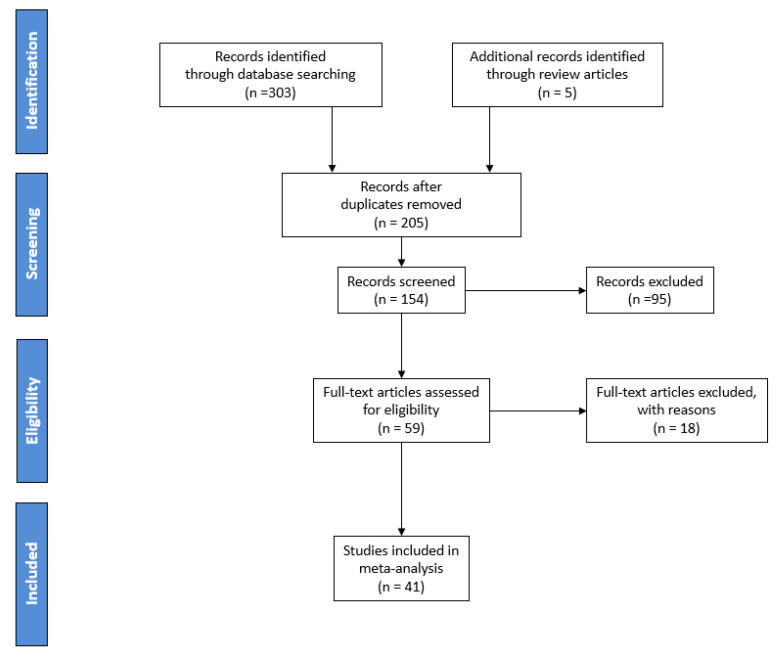
Flowchart of the systematic literature search used in this review.

A comprehensive literature search covering the period from January 2014 to December 2024 was conducted using Scopus and Google Scholar employing the keywords “protein, dairy, milk, cheese, authenticity, adulteration, species, geographical origin” [19,20]. This search yielding over 303 relevant publications. After duplicate removal, 205 unique records remained. Titles and abstracts were screened, resulting in 59 full-text articles assessed for eligibility. Ultimately, 41 studies met the inclusion criteria and were incorporated into the review. Studies were included if they focused on milk authentication using protein- or peptide-based analytical approaches for species or geographical origin differentiation. To narrow the dataset, studies unrelated to authenticity or adulteration through protein analysis were excluded. Specifically, papers utilizing techniques such as gene analysis (e.g., PCR), Raman spectroscopy, near-infrared spectroscopy (NIR), Fourier-transform infrared spectroscopy (FTIR), mid-infrared spectroscopy (MID), and potentiometric methods were omitted. Furthermore, studies addressing adulterants unrelated to species or geographical origin, such as melanin or urea, were excluded to maintain a concentrated scope on protein-based approaches for species differentiation and origin authentication. Finally, papers addressing adulteration in dairy products (e.g., cheese, yogurt, etc.) rather than in milk itself were also excluded, ensuring that the review remained centered specifically on milk authentication. The selection process is summarized in Figure 3, following the PRISMA guidelines for transparent reporting in systematic reviews and reflecting only proteomics-based studies screened for inclusion in this review.

This review provides a critical synthesis of recent advances in milk authentication based on protein and peptide biomarkers, drawing from 41 peer-reviewed studies, 34 focused on intact proteins and 7 on peptides. The primary emphasis is on the detection of species-level adulteration and the fraudulent addition of whey and plant-derived proteins. While various forms of milk fraud—including dilution with water or the addition of compounds such as formaldehyde or urea—remain prevalent, this work concentrates on analytical strategies capable of addressing authenticity issues rooted in protein composition. Drawing upon top-down and bottom-up proteomic methodologies, including LC-MS/MS, MALDI-TOF-MS, electrophoretic, and immunochemical techniques, the review highlights the dual use of intact caseins and whey proteins as well as their enzymatically generated peptides as robust molecular markers. Special emphasis is placed on the integration of these advanced analytical platforms with chemometric workflows—ranging from univariate analyses to multivariate and machine learning models—which enable sensitive and reproducible classification, quantification, and biomarker discovery. By systematically evaluating the past decade of literature, this review underscores the critical role of proteomics-chemometrics convergence in developing reliable, scalable tools for the authentication of milk in increasingly complex global supply chains.

## 2. Economically Motivated Adulteration: Patterns and Implications in Milk from Different Species

The Food and Drug Administration (FDA) defines economically motivated adulteration (EMA) as the intentional and fraudulent substitution or addition of a substance in a product with the aim of enhancing its perceived value or reducing production costs for economic benefit. This practice typically involves one of three approaches: (1) partial or complete substitution of a genuine food ingredient or its valuable component with a cheaper alternative through methods such as addition, dilution, or extension with adulterants; (2) incorporation of minimal amounts of non-authentic substances to conceal the inferior quality of ingredients; or (3) removal or intentional omission of an authentic, high-value constituent without the consumer’s awareness [21].

Various forms of fraud have been reported in the dairy industry, including the addition of vegetable protein, water, whey, or milk from different species as part of economically motivated adulteration. Simple methods such as water dilution increase volume but pose contamination risks, while more sophisticated techniques like the addition of melamine to artificially elevate nitrogen content are significantly harder to detect. Other adulterants such as hydrogen peroxide and formaldehyde are used to prolong shelf life, while substances like vegetable oils, whey, and urea are added to enhance fat and protein content artificially. While some adulterants may pose minimal health risks, hazardous substances such as urea, formaldehyde, detergents, and melamine can have serious adverse health effects, underscoring the need for stringent safety measures and monitoring [22,23,24]. Among these fraudulent practices, species adulteration is one of the most widespread, aimed at reducing production costs and maximizing profits [25].

**Figure 4:**
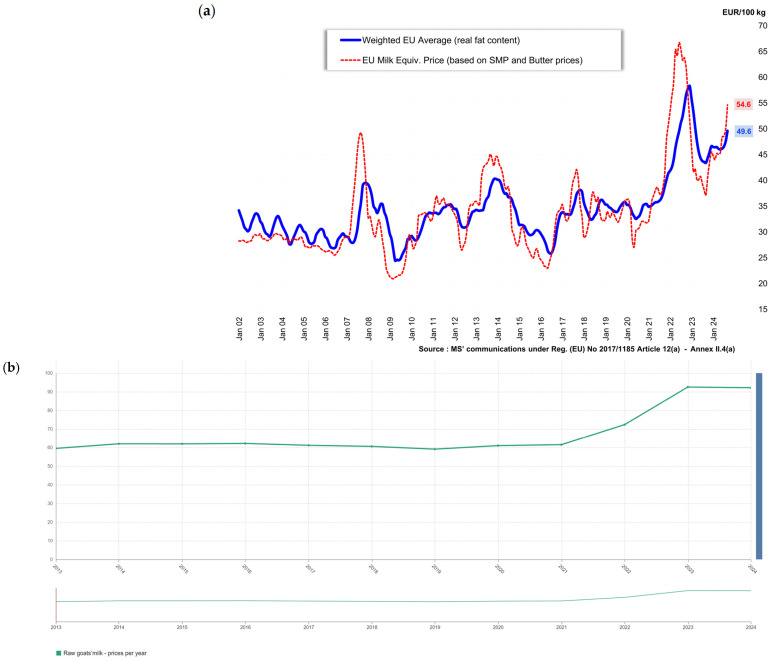

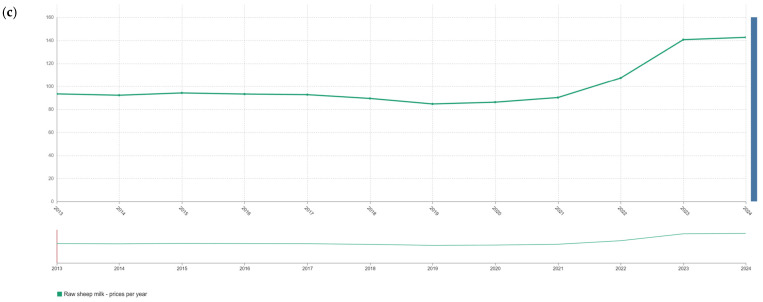
Price trends of cow, goat, and sheep milk, highlighting the economic motivations behind species adulteration in the dairy industry. (**a**) Monthly raw cow milk prices (EUR/100 kg) in the European Union from January 2002 to September 2024, reflecting seasonal variability and moderate long-term increases (source: European Commission) [33]. (**b**) Annual average selling prices of goat milk in Greece from 2000 onward, expressed in euro, and typically 1.5 to 2 times higher than cow milk prices, depending on the year (source: Eurostat, 2025) [34]. (**c**) Annual average selling prices of sheep milk in Greece from 2000 onward, expressed in euro, and consistently the highest among the three, often exceeding cow milk prices by a factor of 2 to 3 (source: Eurostat, 2025) [34].

The price of cow milk, along with other types of milk, exhibits seasonal variation driven by fluctuations in supply, demand, and quality, as documented by the European Commission (Figure 4) and supported by various studies [26,27,28]. Among different milk types, cow milk is generally the least expensive, with the price of goat milk reaching up to twice that of cow milk, while donkey milk can be up to ten times more expensive [29,30,31,32]. Consequently, producers who adulterate higher-value milk with cow milk can achieve substantial economic gains, depending on the type of milk and the extent of adulteration.

The identification of animal species is particularly critical for high-value traditional products, such as cheeses with European Union (EU) designations like protected designation of origin (PDO), protected geographical indication (PGI), or traditional specialty guaranteed (TSG). Notably, over 100 European cheese varieties are classified as PDO or PGI under Regulation No. 1151/2012 of the European Commission [35]. According to the 2023 annual report by the Alert and Cooperation Network of the EU, milk ranks among the top ten foods most frequently subjected to economically motivated adulteration, with 19 cases of fraud reported during the year. Over half of these notifications originated from Cyprus and Greece and were predominantly linked to violations of protected designations of origin (PDO), protected geographical indications (PGI), and traditional specialties guaranteed (TSG) [36]. While indicating the country of origin is generally not mandatory, it is a requirement for PDO products in compliance with Regulation (EU) No. 1151/2012 [37,38].

Milk adulteration presents serious economic and regulatory challenges that extend beyond analytical detection. Regulatory frameworks such as Regulation (EU) No. 1169/2011 on food labeling and Regulation (EU) No. 1151/2012 on geographical indications require precise methods for species identification and traceability, especially for PDO and PGI dairy product. The advanced proteomic and chemometric methods reviewed in the current article, such as LC-MS/MS, MALDI-TOF-MS, and multivariate data analysis, are in alignment with these regulatory restrictions, offering high specificity and reproducibility, supporting both product authentication and regulatory audits. From an economic view, although these technologies require further costs in equipment, software, and personnel training, the long-term financial benefits are substantial. Food fraud can result in recalls, fines, legal liabilities, and reputational damage, all of which are far costlier than prevention measures. Moreover, reliable authentication protects the premium valued milks (e.g., buffalo, donkey, and camel) and enhances consumers’ trust. Cost–benefit analyses have shown that investing in robust analytical platforms reduces vulnerability to fraud and enhances market stability and brand equity [39,40,41,42,43]. Thus, the adoption of advanced milk authentication methods is not only scientifically justified but also economically beneficial.

## 3. Analytical Strategies for Milk Authentication: From Electrophoresis to Proteomics

While the present review is dedicated to protein- and peptide-based approaches for milk authentication, it is important to acknowledge that alternative methodologies—such as DNA-based techniques, spectroscopic platforms (e.g., NIR, FTIR, and Raman), and biosensor technologies—also play significant roles in the broader field of dairy fraud detection. These complementary methods are briefly referenced in this section for contextual completeness and to highlight methodological diversity. However, they were not included in our systematic literature review and are not evaluated in depth. Readers seeking detailed appraisals of these approaches are directed to specialized reviews focused on molecular diagnostics, vibrational spectroscopy, and sensor-based authentication.

It is essential for both the dairy industry and consumers to have reliable methods to distinguish between authentic and adulterated milk and dairy products. Various techniques are utilized to detect species adulteration in milk and dairy products, each with distinct advantages and limitations. Current methodologies for species authentication primarily rely on protein and DNA markers and can be classified into four main categories: protein-based methods, spectroscopic methods, DNA-based methods, and (bio)sensors.

**Electrophoretic techniques** are inexpensive and efficient but can produce ambiguous results due to overlapping bands. They are also unsuitable for quantifying processed products or detecting adulteration involving heat-treated whey proteins, and they require reference standards for certain methods like IEF [44,45]. Two-dimensional gel electrophoresis (2D-PAGE) combines IEF and SDS-PAGE to separate proteins first by isoelectric point and then by molecular weight, allowing the resolution of thousands of protein spots in complex mixtures [46]. Some of the earliest studies in protein-based milk authentication include Wolfgang et al. and Anguita et al. (1997), who utilized ELISA to detect cow casein in goat and ewe milk, laying the groundwork for protein-based authenticity detection [47,48]. Similarly, Mayer et al. employed IEF to determine the percentages of cow, ewe, and goat milk in cheese, further advancing the development of protein-based analytical methods [49].

**Immunochemical techniques** are highly sensitive, fast, and easy to implement in routine analyses. However, they may generate false positives due to cross-reactivity and false negatives if the samples have undergone processing, and their effectiveness depends on the availability of specific antibodies [25,50].

**Chromatography coupled with mass spectrometry** is highly sensitive and specific, allowing quantitative and multiplex analyses. Despite these advantages, it requires high-end instrumentation, trained personnel, and advanced bioinformatics workflows. MS-based milk authentication typically relies on search engines such as Mascot and PEAKS Studio for peptide identification, along with databases such as UniProt for protein annotation. Tools like UniPept offer taxonomic classification of tryptic peptides, while BLAST (version 2.16.0) and HMMER (version 3.4) are employed for sequence alignment and homology assessment. In recent years, open-access proteomics repositories such as PRIDE, ProteomeXchange, and PeptideAtlas have improved data reproducibility and cross-study validation in food proteomics by enabling the reuse of annotated MS/MS data and metadata [51,52,53,54,55,56,57,58,59,60]. Spectroscopy offers speed, simplicity, and high-throughput capability and is often non-destructive, with some techniques allowing portability [61]. However, it relies on large databases and chemometric analysis and may involve expensive equipment depending on the method [62,63]. A significant early application of mass spectrometry in milk authentication was conducted by Angeletti in 1998, using MALDI-MS to detect the addition of bovine milk to buffalo milk intended for mozzarella cheese production [64]. Recent innovations have further enhanced the use of proteomics in milk authentication. For instance, Karamoutsios et al. (2025) combined MALDI-TOF MS analysis of casein proteins with artificial neural networks to classify bovine, goat, and sheep milk samples with high precision. This study highlights the growing role of machine learning in improving the efficiency and reliability of mass spectrometry-based authentication methods [65].

**DNA-based approaches** like PCR-RFLP and species-specific PCR are simple and highly specific but lack quantification because it is difficult to accurately determine the amount of milk mixed in since the content of DNA in the milk is not fixed adding to the possibility of degradation during processing [66]. Real-time PCR, on the other hand, is quantitative, highly sensitive, specific, and suitable for multiplex analyses, though it requires moderately expensive equipment [67]. Notably, Plath et al. (1997) conducted one of the pioneering studies employing DNA-based techniques for species identification [68]. Finally, biosensors provide a rapid, cost-effective, and user-friendly solution with the potential for portability, but they often deliver qualitative results and can be limited by sensitivity [69].

On the other hand, isotope and elemental fingerprinting are widely used for geographical origin authentication and have demonstrated consistent accuracy in verifying the provenance of dairy products [70]. Other chemical and physical methods, such as near-infrared spectroscopy (NIR) and nuclear magnetic resonance (NMR), show promise but require further studies with larger sample sizes to better evaluate their discriminative capabilities [71]. Emerging DNA-based techniques such as metabarcoding have shown potential for origin authentication; however, metagenomics, which provides a more comprehensive view of cheese microbiota at the strain level, offers greater resolution. Combining methods targeting different molecular or chemical markers may further enhance accuracy and reliability in this field [72].

Table 1 presents a comparative summary of key analytical techniques applied in milk authentication, highlighting their respective strengths, limitations, and suitability for specific use cases.

## 4. Protein Biomarkers for Detecting Milk Adulteration

Milk proteins have been extensively studied, with numerous bioactive proteins and peptides identified, while many others remain unexplored. Caseins—namely αs1-casein, αs2-casein, β-casein, and κ-casein—account for the largest fraction of milk protein, with their concentrations varying across different animal species. Figure 1 illustrates the relative concentrations (g L^−1^) of caseins in milk across different species. In addition to quantitative values, the figure includes relevant molecular characteristics. The notation “CN” refers to the casein protein, while the number followed by “P” indicates the degree of phosphorylation of the isoform (e.g., αs1-CN 9P denotes αs1-casein with nine phosphate groups). Each casein type is also annotated with its known genetic variants (e.g., A–H for αs1-casein), which represent allelic polymorphisms identified in different species and breeds. These variants can affect protein expression, micellar structure, and overall milk functionality.

Whey proteins include α-lactalbumin, β-lactoglobulin, bovine serum albumin, lactoferrin, and immunoglobulins, as illustrated in Figure 2. In addition to these groups, milk contains non-protein nitrogen compounds and over 120 milk fat globule membrane proteins, accounting for 1% to 2% of total milk proteins, along with more than 70 enzymes and hormones, highlighting its complexity and substantial nutritional significance [6,9]. Figure 2 also includes structural annotations of α-lactalbumin and β-lactoglobulin, indicating known glycosylation patterns and common allelic variants (e.g., A–C for α-lactalbumin; A–J and W for β-lactoglobulin), which reflect interspecies genetic polymorphisms with implications for stability, digestibility, and processing behavior.

Importantly, both genetic variants and post-translational modifications such as phosphorylation can alter a protein’s mass, charge, and conformation, affecting its performance in electrophoretic separation, enzymatic digestion in bottom-up proteomics, and ionization efficiency in MS-based workflows. Such variations may either improve or complicate the specificity of biomarker detection, depending on the analytical platform used [6].

These bioactive components may exist either as intact proteins or as peptides encrypted within the primary sequences of milk proteins [73]. For instance, databases like BomiProt 2.0 catalog 7459 proteins identified in cow milk [74], and the “Portail Data INRAE” online repository lists 4654 cow milk proteins [75]. Additionally, species-specific bioactive peptides have been systematically compiled, as demonstrated in the comprehensive work of Nielsen et al. (2017) [76]. For most commercially significant milk species, protein sequences are well-documented in repositories such as UniProt and NCBI. However, variations due to polymorphisms, post-translational modifications, or geographical factors remain incompletely mapped, particularly for specific breeds or less-studied wild species [77,78,79,80]. Furthermore, not only do these proteins vary due to genetic and environmental factors, but their concentrations also differ substantially among species, reflecting unique physiological adaptations.

The protein content in milk varies significantly among species, ranging from approximately 1% to 20%. This variation is intricately linked to the growth rates and essential amino acid requirements of the neonates specific to each species. As mentioned before, milk comprises two primary protein groups: caseins and whey proteins. However, the ratio of these proteins varies widely across species (Table 2), reflecting genus- and species-specific adaptations to neonatal nutritional and physiological needs [4]. The distinct protein profiles of casein and whey proteins in milk serve as a unique molecular “fingerprint” for each species. These profiles are highly valuable in detecting adulteration, as they can be accurately characterized using advanced analytical techniques [9].

Casein is a term that was derived from the Latin word caseus, meaning cheese [82]. Caseins are fundamental to milk functionality, primarily due to their ability to form colloidal micelles. These micelles solubilize calcium and phosphate, thereby preventing calcification in the mammary gland. Despite extensive research, the structure of casein micelles remains debated, with studies focusing on their size, composition, and reconstitution [83].

**Figure 5:**
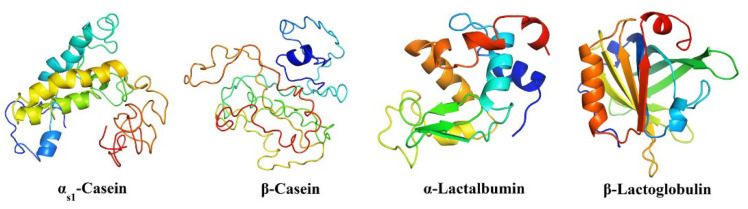
Three-dimensional structures of the major proteins in cow’s milk. Reproduced with permission from Pan et al. (2020) [86].

Caseins exhibit significant interspecies variability, particularly in the α-casein fraction. For instance, αs1-casein in goat milk varies widely (0–26% of total casein), impacting milk’s coagulation properties and the quality of cheeses derived from sheep and goat milk [4,81,84]. Structurally, caseins are rich in apolar amino acids, contributing to their low solubility in water. However, this is offset by their high phosphate content, low sulfur amino acid levels, and, in κ-casein, substantial carbohydrate content, which collectively enhance solubility under specific conditions. Caseins are also highly proline-rich, which disrupts the formation of α-helices and β-sheets (Figure 5), rendering them susceptible to proteolysis without prior denaturation [83,85]. These characteristic aids digestibility and facilitate hydrolysis during cheese production, contributing to flavor and texture development [81].

Caseins exhibit significant inter-species diversity [87]. While the two principal whey proteins, α-lactalbumin and β-lactoglobulin, display a high degree of similarity across species, the caseins are far more variable. The αs-casein fraction, for instance, differs markedly between species. Human milk lacks αs-casein entirely, while horse and donkey milk exhibit highly heterogeneous α-caseins. Conversely, all studied species contain a β-casein with electrophoretic mobility similar to bovine β-casein, though the sequenced β-caseins show low similarity. Human and equine β-caseins occur in multi-phosphorylated forms, further contributing to this diversity. κ-casein, essential to the casein system, is present in all studied species but exhibits significant variations. Human κ-casein, for instance, is highly glycosylated, containing 40–60% carbohydrate compared to approximately 10% in bovine κ-casein. This carbohydrate occurs as diverse and complex glycosideic residues of proteins distinct from those in bovine milk [4,5,81].

**Figure 6:**
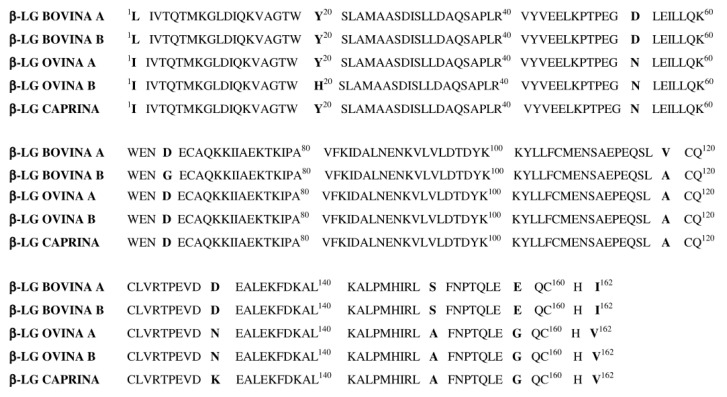
Amino acid sequence of bovine, ovine, and caprine β-lactoglobulin. Superscript numerals (e.g., ^20^, ^40^, ^60^, etc.) indicate the residue number of the terminal amino acid shown in each line segment, facilitating alignment with known bioactive peptide fragments (e.g., f(15–20), f(102–105)) discussed in the literature.Reproduced with permission from Hernández-Ledesma et al. (2008) [89]. © Springer-Verlag 2007.

Whey proteins constitute approximately 20% of total protein in bovine milk and include β-lactoglobulin (β-lg), α-lactalbumin (α-la), and blood serum albumin (BSA), along with trace proteins like lactoferrin, serotransferrin, and various enzymes. These proteins are structurally conserved across species, with sheep, goat, and buffalo milk exhibiting compositions similar to bovine milk. β-lactoglobulin, the most abundant whey protein, has a highly ordered structure comprising 10–15% α-helix, 43% β-sheet, and 47% unordered elements, forming a compact, globular shape characterized by a β-barrel or calyx (Figure 6). In contrast, α-lactalbumin, which constitutes 20% of bovine whey proteins, is a smaller globular protein. It features a structure of 26% α-helix, 14% β-sheet, and 60% unordered elements and shares notable conformational similarities with lysozyme [81,88].

### Survey of Proteomic Studies on Milk Authentication Using Protein Biomarkers

**Figure 7:**
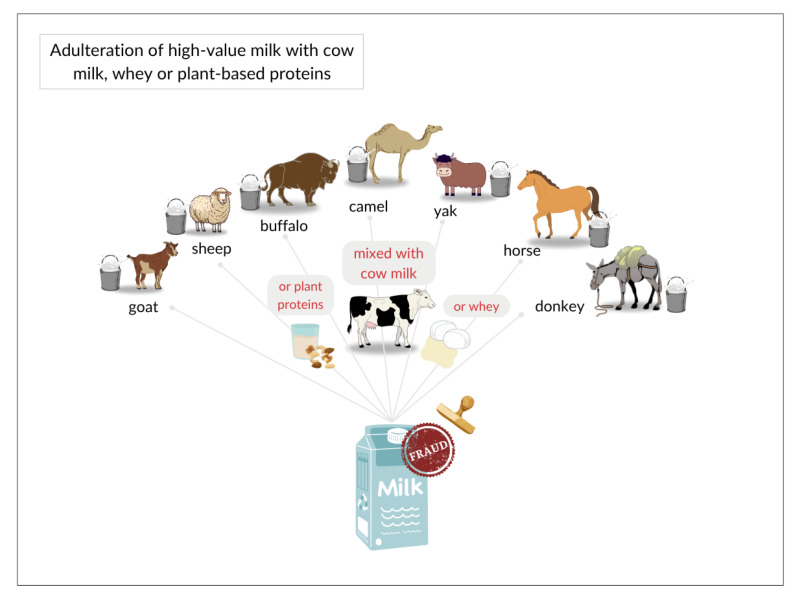
Schematic representation of common adulteration practices in high-value milk using cow milk, whey, or plant-based proteins.

In this review, we critically evaluated 41 peer-reviewed studies published between 2014 and 2024 that employed proteomic methodologies to investigate milk adulteration. Among these, 34 studies specifically targeted protein-based markers to detect fraudulent practices. These studies reported instances of interspecies adulteration, including goat, sheep, buffalo, camel, yak, horse, and donkey milk being adulterated with cow milk as well as cases where cow milk was adulterated with plant-derived proteins or whey cheese (Figure 7). These practices are often considered more “natural” adulteration methods due to their similarity to authentic milk components, but they can still compromise nutritional value, allergenicity, and consumer trust.

In Table S1, we summarize these studies, highlighting the specific milk proteins as indicators of adulteration in each case. This compilation aims to provide a comprehensive overview of how milk proteins are used in species differentiation and fraud detection. To enhance interpretability, the proteins are categorized into caseins, whey proteins, and others, illustrating their distinct roles as molecular markers in milk authentication.

Before delving into the analysis of individual milk proteins and their applications in detecting adulteration, it is essential to outline the methodologies employed across various studies. Protein analysis using mass spectrometry (MS) typically follows two principal approaches: top-down and bottom-up strategies. The top-down approach involves the direct analysis of intact proteins, most commonly via electrospray ionization mass spectrometry (ESI-MS) [90]. In studies such as those by Tehrani et al. (2024) and Sassi et al. (2015), proteins are identified based on their molecular weight (M_r_) [16,91]. For instance, according to UniProt, the theoretical molecular weight of α-lactalbumin (α-La) differs across species: 14,186.0 for cow milk, 14,194.0 for goat milk, and 14,255.0 for sheep milk. These molecular weight differences allow for species-specific differentiation of the α-La protein in these studies. Top-down approaches using ESI-MS have also been applied to other species such as goat, sheep, and buffalo milk. These methods allow the direct detection of intact protein isoforms and their mass shifts, facilitating species differentiation and authentication based on casein and whey protein profiles.

Conversely, the bottom-up approach involves enzymatic digestion of proteins into peptides, which are subsequently analyzed by MS. This method has been employed in studies by Ji et al. (2023), Fan et al. (2023), and Ji et al. (2024), which focus on peptide-level identification using peptides generated through tryptic hydrolysis [18,92,93]. While these studies begin with peptide analysis, the use of advanced bioinformatics tools and protein databases allows for identification at the protein level. This approach underscores the significance of peptide analysis in enabling accurate protein-level identification.

In protein-based milk authentication, the most widely adopted approaches involve chromatographic techniques coupled with mass spectrometry, such as LC-MS/MS and HPLC-QTOF [18,92,93,94], due to their high sensitivity and multiplexing capacity. Depending on the proteomic strategy, QTOF platforms are applied either in bottom-up approaches—where peptides are analyzed following enzymatic digestion—or in top-down workflows for intact protein analysis. MALDI-based workflows, particularly when combined with machine learning, have also shown promise in several recent studies [16]. This section focuses on the application of mass spectrometry-based proteomics in milk authentication, following the comparative overview of conventional electrophoretic and immunochemical techniques presented earlier.

The complete list of protein biomarkers identified in the reviewed studies, along with their detection method, milk origin, and analytical details, is provided in Supplementary Table S1. Key biomarkers with high sensitivity or novel applications are highlighted in the text below.

First, among caseins, α-casein stands out as a versatile and widely studied biomarker; it was used in 10 studies as a biomarker. It has been utilized to identify adulteration with cheese whey in cow milk [95], cow milk in sheep [96], donkey [32], and horse milk [18,92] as well as soybean milk in cow milk [97]. Notably, α-casein has proven sensitive enough to detect adulteration at concentrations as low as 0.1% cow milk in horse milk, underscoring its efficacy in complex matrices such as milk. Additionally, αs1-casein plays a crucial role as a marker in allergen detection due to its allergenic properties This makes camel and donkey milk, which lack αs1-casein, preferred hypoallergenic alternatives for infants and individuals with cow milk protein allergies [32,98].

Similarly, β-casein has been studied in 10 investigations as a biomarker for detecting various forms of milk adulteration These include the presence of cow milk in yak [99], goat [16,91], sheep [16], horse [92], and donkey milk [32] as well as adulteration with cheese whey in cow milk [95]. While β-casein demonstrates a broad range of applications across diverse adulteration scenarios, its detection limits are generally higher than those of α-casein for specific adulterants. Nevertheless, as reported by Ren et al. (2014), β-casein remains an effective biomarker for detecting cow milk in yak milk, with its performance unaffected by common processing treatments such as heating, acidification, and rennet addition [99].

In contrast, γ-casein was studied in only one investigation, as it is not a standalone protein but rather a fragment of β-casein generated through plasmin hydrolysis. The resulting C-terminal peptides—γ-caseins—are defined as γ1: β-CNf29–209; γ2: β-CNf106–209; and γ3: β-CNf108–209 [4,100]. Despite limited research, γ-caseins are recognized for their utility in adulteration detection. The isoelectric focusing of γ-caseins following plasminolysis is an established method for identifying cow milk in sheep, goat, and buffalo milk, as outlined in European Union regulations (European Union, 2008b) [101]. Finally, κ-casein plays a significant role in identifying adulteration in goat, donkey, and horse milk with cow milk, particularly in immunoassay-based techniques [102,103]. Its exceptional sensitivity is noteworthy, with studies reporting the detection of adulteration at levels as low as 0.04% cow milk in goat milk when combined with immunoassays, such as in the method presented by Angelopoulos et al. (2015) [102]. Immunoassays are widely used due to their simplicity, rapid analysis, and ability to efficiently screen large numbers of samples, with some formats being adaptable for field testing. However, these methods heavily rely on specific antibodies, which limits their applicability to novel or unexpected protein modifications. Despite this limitation, κ-casein remains one of the most sensitive and reliable biomarkers. It is widely employed in current methods for milk adulteration detection.

Among whey proteins, α-lactalbumin has been identified as a biomarker in seven studies, primarily for detecting cow milk in goat [16], sheep [16], buffalo [91,104], donkey, and horse milk [18,92] as well as soybean milk in cow milk [97]. α-Lactalbumin demonstrates high sensitivity, with the study by Ji et al. (2023) [18] detecting adulteration levels as low as 0.1% cow milk in horse milk. However, Ji et al. (2024) [92], observed reduced thermal stability, with detection sensitivity dropping to 1% cow milk in pasteurized horse milk. These findings highlight α-lactalbumin’s limitations under heat treatment, making it less reliable for thermally processed samples.

Lastly, β-lactoglobulin has been widely used to detect cow milk adulteration in goat [93], camel [94], buffalo [91,98], and horse milk [18,92]. Although its range of applications is slightly narrower than α-lactalbumin, it is highly effective, achieving detection limits as low as 0.1% cow milk in goat milk [93].

In addition to casein and whey proteins, other proteins have also been reported in the literature as potential biomarkers. These include immunoglobulin G [105,106,107], lactoferrin [108,109], and glycomacropeptide (GMP) [45,110,111]. Although these proteins are less abundant and less widely studied compared to casein and whey proteins, their distinctive properties may provide valuable insights for detecting milk adulteration in specific applications.

## 5. Peptide Biomarkers in Milk Authentication

Peptide analysis is increasingly favored over direct protein analysis in proteomic approaches for milk and dairy authenticity studies due to several key advantages. Table 3 provides a comparative overview of the total number of amino acids and molecular weights of major caseins in five key milk-producing animals. These interspecies differences in primary structure form the molecular basis for identifying unique, species-specific peptide markers. Unlike intact proteins, peptides can be screened for species-specific sequences, which are more distinct and easier to differentiate, particularly in complex matrices such as milk and dairy products [112,113]. Enzymatic digestion further simplifies the analytical workflow by breaking down complex protein structures into smaller, more stable peptides, thereby mitigating challenges associated with protein folding and post-translational modifications [114,115]. Moreover, peptides demonstrate greater stability under industrial processing conditions, including heat treatment and fermentation, making them particularly suitable for analyzing dairy products. Their stability also ensures more consistent signal generation in mass spectrometry, enhancing both sensitivity and reproducibility [112,116]. Additionally, peptide-level analysis facilitates high-throughput workflows and enables seamless matching with theoretical sequences in databases like UniProt, streamlining the identification and quantification processes [115,117]. Collectively, these attributes underscore the superiority of peptide analysis in achieving precise, reliable, and reproducible results in milk and dairy protein characterization and authentication.

From Hassanin et al. (2022) study, we observed notable variations in the amino acid sequences and molecular weights of the four major caseins—α-S1-casein (CSN1S1), α-S2-casein (CSN1S2), β-casein (CSN2), and κ-casein (CSN3)—among five key dairy animal species. For instance, α-S1-casein in Arabian camels contained 222 amino acids with a molecular weight of 25.843 kDa, whereas goats exhibited a slightly shorter sequence of 213 amino acids and a molecular weight of 24.13 kDa. These species-specific variations reflect evolutionary adaptations and may influence the functional properties of caseins, such as their digestibility, bioactivity, and structural organization in milk micelles. The comprehensive comparison presented in Table 4 underscores the value of phylogenetic and bioinformatics analyses [118].

An essential step in proteomic approaches that utilize signature peptides as markers is their careful selection. Across the reviewed studies, the primary objective was to identify peptides with high specificity, stability, and robustness for differentiating milk types. While the foundational principles were consistent, variations in detailed criteria and methodological approaches reflected the diversity of research goals and analytical techniques.

Tryptic digestion was universally employed as the primary enzymatic method for peptide generation due to its efficiency and precision in cleaving at lysine and arginine residues, producing consistent and analyzable fragments [90]. Notably, Nardiello et al. (2018) employed a multi-enzyme digestion strategy using both trypsin and chymotrypsin to enhance sequence coverage and improve peptide identification, distinguishing it from the other studies [116]. Specificity validation was conducted through BLAST searches in databases such as UniProt and NCBI, ensuring the uniqueness of selected peptides to their respective milk proteins and species [66,114,115]. Casein, a highly abundant protein with species-specific differences, was consistently prioritized across studies, underscoring its role as a reliable marker for milk authenticity analysis.

Furthermore, stability and integrity of peptides were emphasized, with most studies evaluating resistance to thermal degradation and processing effects such as heating, pressure, and fermentation [112,114]. Zhang et al. (2024) specifically assessed peptide stability under these processing conditions and incorporated it as a key validation step [112]. Across all studies, peptides prone to dynamic modifications or with missed cleavage sites were systematically excluded to ensure clean digestion products and reliable quantification [114,115]. Peptide length was another critical consideration, with Zhang et al. (2022) recommending an optimal range of 8–20 amino acids to enhance detectability and ensure consistent retention times [66].

Despite these shared principles, notable differences emerged in the specific selection and validation steps across the reviewed studies. Hao et al. (2022) introduced the DiSta principles, focusing on peptides with high digestibility and stability that reach peak concentration within 1–2 h post digestion and remain stable for at least 24 h [114]. Similarly, Ke et al. (2017) incorporated isotopically labeled peptides to enhance quantification accuracy and correct for matrix effects, a methodological refinement that was not emphasized in other studies. They also uniquely utilized Biolynx software for computational peptide prediction and excluded peptides prone to oxidation, prioritizing those with high MS signal intensity and reproducibility in sample preparation [115]. Lastly, Zhang et al. (2024) distinguished their methodology by integrating multiple reaction monitoring (MRM) for precise quantification, making their approach particularly robust for complex sample matrices [112].

In peptide-based milk authentication, liquid chromatography coupled with tandem mass spectrometry (LC-MS/MS) has emerged as the predominant analytical approach owing to its high specificity, sensitivity, and compatibility with enzymatic hydrolysis workflows [66,112,113,114,115,116]. These methods enable accurate detection of species-specific peptide markers and are well-suited to both targeted and untargeted peptidomic strategies. In contrast, MALDI-based mass spectrometry has only recently been introduced in this field and remains underexplored, with its use limited to a small number of studies focused primarily on rapid peptide profiling. The current methodological landscape thus reflects a clear preference for high-resolution, quantitative LC-MS/MS platforms, while the potential of MALDI-based approaches warrants further investigation.

### Application of Peptide Biomarkers in Milk Adulteration Studies

Studies focused on milk products were limited to five papers (Table 4), highlighting the need for further exploration in this area. To facilitate the identification of the specific peptides listed in Table 4, an index table (Table 5) was created. This index provides a comprehensive overview of the peptide sequences, their corresponding marker proteins, and references to the respective studies.

These studies identified peptide biomarkers linked to major milk proteins, including αs1-casein, αs2-casein, β-casein, κ-casein, β-lactoglobulin, and α-lactalbumin, as well as numerous uncharacterized peptides. Among these, αs2-casein emerged as the most frequently reported source of biomarkers, although other casein proteins, such as αs1-casein, β-casein, and κ-casein, were also found to contain numerous species-specific peptide markers across all studied species.

Peptides originating from αs1-casein included FFVAPFPEVFGK (38–48) in cow milk [114,115] and FVVAPFPEVFR [115] in goat and sheep milk, differing by the last two amino acids. Similarly, Zhang et al. (2022) and Lu et al. (2023) [40,101] reported the peptide HQGLPQEVLNENLLR [66,117], a sequence that consistently emerged as a robust biomarker for cow milk.

Zhang et al. (2024) detected the peptides NMAINPSK in cow milk and NMAIHPSK in buffalo milk, which differed by a single amino acid substitution in αs2-casein protein, yielding species-specific peptides [112]. Additionally, FALPQYLK, derived from αs2-casein, was found in the studies of Hao et al. (2022) and Ke et al. (2017) as a robust marker for cow milk, reinforcing its importance in milk authentication workflows [114,115].

β-casein contributed significantly to the peptide biomarkers, with sequences such as SPAQTLQWQVLPNTVPAK in goat milk [66,112], closely resembling SPAQTLQWQVLPNAVPAK in sheep milk and SPAQILQWQVLPNTVPAK in buffalo milk [112]. Additionally, the peptide NAVPITPTLNR (131–141) in cow milk [115,117] was identified alongside NAGPFTPTVNR (131–141) in sheep and goat milk [115], demonstrating evolutionary conservation while offering specific markers for species differentiation.

In addition to caseins, κ-casein peptides offered valuable markers for species differentiation. Zhang et al. (2024) identified the peptide SCQAQPTTMAR in cow milk, while SCQDQPTAMAR, which differed by two amino acids, was specific to sheep milk [112].

Finally, α-lactalbumin yielded peptides such as LAFNPTQLEGQCHV (167–180) in goat and sheep milk [115] compared to LSFNPTQLEGQCHI in yak milk [112]. The peptide HQGLPQEVLNENLLR, although primarily associated with αs1-casein, also exhibits overlap with sequences derived from α-lactalbumin, further underscoring its utility as a widely applicable marker across multiple studies.

These findings highlight the evolutionary conservation of milk protein sequences across species while also revealing subtle, species-specific variations that serve as reliable biomarkers. Such markers are critical for milk authentication, particularly in complex matrices where distinguishing between closely related species is challenging. The consistency of these biomarkers across multiple studies underscores the potential of peptide-level analysis in proteomic studies, offering both sensitivity and specificity for detecting milk adulteration and ensuring product authenticity. However, in addition to these well-characterized peptides, many studies also report the presence of uncharacterized peptide markers. A significant number of these remain structurally undefined, presenting notable analytical challenges. Without confirmed sequence identity or clear protein origins, it becomes difficult to confidently link such peptides to specific species or milk proteins. This uncertainty limits reproducibility across laboratories and analytical platforms and may compromise the comparability of results. Furthermore, these unknown peptides may be influenced by sample handling, processing conditions, or enzymatic activity, further complicating their interpretation. To enable their reliable integration into authentication workflows, future studies should prioritize structural elucidation using MS/MS fragmentation, spectral library matching, and orthogonal validation techniques.

## 6. Chemometric Workflows for Interpreting Proteomic Data in Milk Authentication

The authentication of milk based on protein and peptide markers has advanced considerably over the past decade, largely due to the integration of high-resolution proteomic techniques with robust chemometric analysis. In this context, chemometric tools are not merely supportive but form the analytical backbone of proteomics-based milk authentication. The complexity of milk proteomic data—particularly from mass spectrometry platforms such as MALDI-TOF-MS and LC-MS/MS—demands the use of multivariate statistical methods capable of handling high dimensionality, biological variability, and analytical noise. Alongside these approaches, univariate statistical analyses continue to play a critical role, particularly in targeted workflows using platforms such as ELISA, capillary electrophoresis, lab-on-a-chip devices, or biosensors. These methods often apply linear regression, ANOVA, or sigmoidal dose–response curve fitting to quantify species-specific protein markers or peptide concentrations.

In this context, chemometrics is not simply about selecting statistical models; it involves designing a comprehensive workflow encompassing every stage of the process—from raw data acquisition to meaningful biological interpretation. In milk authentication, this process is typically aimed at detecting adulteration or verifying species origin, both of which require reliable discrimination based on proteomic data. This section provides an overview of the chemometric workflows most commonly applied in proteomics-based milk authentication studies. It distinguishes between fingerprinting approaches, which generate global protein profiles and typically rely on unsupervised methods for pattern recognition and class separation [16,119] and protein or peptide quantification strategies (both targeted and untargeted), which are better-suited to supervised learning techniques for sample classification and biomarker selection [94,104,111].

A structured overview of these chemometric workflows is presented, highlighting the key steps from data preprocessing to multivariate modeling.

In milk authentication, chemometric tools are not merely used for data reduction or visualization—they serve as powerful analytical engines that translate complex proteomic profiles into actionable insights. Unsupervised techniques such as principal component analysis (PCA) are typically employed in the exploratory phase to reveal clustering tendencies and underlying data structure, particularly within high-dimensional peptide datasets [120]. In contrast, supervised models like partial least squares–discriminant analysis (PLS-DA) or linear discriminant analysis (LDA) are utilized to enhance class separation and improve predictive accuracy—an essential requirement when distinguishing between closely related species or detecting low-level adulteration [121].

Beyond algorithmic implementation, the rationale for these methods lies in their ability to convert detailed spectral or chromatographic information into robust classification systems. However, strong performance on training data does not guarantee real-world applicability. Therefore, model validation—through techniques such as k-fold cross-validation or the use of independent test sets—is crucial to confirm generalizability and mitigate overfitting [122]. When embedded in well-structured workflows and combined with high-resolution mass spectrometry data, these chemometric strategies provide a reproducible and scalable solution for routine milk authenticity assessment.

### 6.1. Data Preprocessing Strategies in Proteomics-Based Milk Authentication

In proteomics-based milk authentication, data preprocessing is a foundational step that significantly influences the reliability, comparability, and interpretability of chemometric models. Mass spectrometry data often contain noise, systematic biases, and biological variability that must be corrected prior to analysis. Effective preprocessing ensures that the resulting data reflect true biological differences and that downstream modeling is both valid and robust. Standard preprocessing pipelines include smoothing (e.g., Savitzky–Golay filter), baseline subtraction, peak alignment, and normalization. These steps correct technical variation and improve data comparability across samples. [122].

In MALDI-TOF-MS applications, Savitzky–Golay smoothing is commonly used to reduce high-frequency noise while preserving peak shape [123,124,125], whereas LC-MS/MS workflows often require correction for retention time shifts and background variability. Software tools such as ClinProTools (version 2.2) (for MALDI) and MaxQuant (version 2.0.3.0) (for LC-MS/MS) offer built-in algorithms for peak processing, with downstream statistical analysis typically performed in Perseus [18,91,92].

These platforms implement essential functions including peptide filtering, retention time alignment, and intensity normalization, though reporting of these steps varies widely across studies [18,92,93].

A representative example is the study by Tehrani et al. (2024), which applied a complete MALDI-based preprocessing workflow to authenticate milk species. The authors performed baseline correction and smoothing in ClinProTools, followed by peak ratio normalization, which significantly reduced relative standard deviation (RSD) and enhanced class separation. After preprocessing, the data were log-transformed and z-score scaled, improving variance homogeneity and model stability in PCA and PLS-DA [16]. Likewise, Yang et al. (2019) demonstrated that log transformation and standardization are critical for uncovering latent structure in unsupervised multivariate models [126].

Together, these studies underscore that preprocessing is not a standardized routine but a methodologically critical phase that must be tailored to the proteomic platform, experimental design, and statistical goals. The rigor and transparency of preprocessing decisions are fundamental to ensuring reproducibility and validity in milk authentication research.

### 6.2. Feature Selection and Exploratory Analysis: Identifying Discriminative Biomarkers

Following data preprocessing, feature selection and exploratory analysis play a pivotal role in proteomics-based milk authentication workflows. These steps are essential for reducing dimensionality, identifying informative variables, and revealing patterns or sources of variance prior to supervised classification [120].

Feature selection is performed to identify the most discriminatory proteins or peptides for differentiating between milk samples of different species or adulteration levels. Univariate filtering is often applied as an initial step, relying on statistical criteria such as *p*-values derived from ANOVA, *t*-tests, or non-parametric tests like the Wilcoxon rank-sum test. Studies employing LC-MS/MS workflows, such as those by Ji et al. (2023, 2024) and Fan et al. (2023), consistently used one-way analysis of variance (ANOVA) with significance thresholds set at *p* < 0.05, often in conjunction with fold-change cut-offs (typically |FC| ≥ 2), to isolate differentially abundant proteins [18,92,93]. These variables were further visualized using volcano plots, which provided an integrated view of statistical significance and magnitude of expression changes, facilitating the prioritization of candidate markers. In MALDI-TOF-MS applications, such as the study by Sassi et al. (2015), combining the Wilcoxon test, Anderson–Darling test, and *t*-test under highly stringent *p*-value criteria (*p* < 1 × 10^−6^) ensures high confidence in the selection of peptides or proteins showing a statistically significant difference in signal intensity or mass value [91].

Univariate statistical filtering was also widely employed in studies using alternative analytical platforms. For example, Chi et al. (2024) applied *t*-tests and fold-change analysis to ELISA-derived HPLC peak areas to identify β-lactoglobulin as a species-specific marker [127]. Trimboli et al. (2017, 2019) used CE to quantify cow milk adulteration in ewe and buffalo milk and performed one-way ANOVA with Bonferroni post hoc tests, alongside normality assessments, to validate their detection strategy [96,104]. Similarly, Yasmin et al. (2020) used SDS-PAGE to evaluate protein profiles across animal species, applying ANOVA followed by Tukey’s HSD to determine statistically significant differences in band intensity [98]. Santos et al. (2023) utilized lab-on-a-chip capillary electrophoresis and employed Dunnett’s test to compare protein peak areas in control versus adulterated samples [95]. In an immunoassay-based study, Ren et al. (2014) applied linear regression and reported statistically significant group differences in ELISA response values [99]. Lastly, Zhang et al. used *t*-tests and fold-change interpretation to assess fluorescence sensor array data for species identification [97].

Although univariate filtering was the dominant strategy for variable reduction, multivariate feature selection techniques were employed in select studies—most notably in fingerprinting-based workflows. Multivariate feature selection methods are also commonly employed, particularly when dimensionality reduction is required for classification modeling. Partial least squares–discriminant analysis (PLS-DA) provides variable importance in projection (VIP) scores, which quantify the contribution of each variable to the model’s discriminatory power. Features with VIP scores above a predefined threshold (e.g., VIP ≥ 1.0) are typically retained for further analysis. In the study by Sassi et al. (2015), genetic algorithms (GA) were used to optimize feature subsets for model construction, iteratively selecting combinations of spectral peaks that maximized classification accuracy. These algorithms provided a powerful means of navigating high-dimensional data by identifying variable sets with strong discriminatory potential [91].

Prior to applying supervised models, unsupervised exploratory methods are used to investigate inherent data structure, assess biological variability, and detect batch effects or outliers. Principal component analysis (PCA) is the most widely used method in this context. PCA has been employed in multiple studies (e.g., Ji et al., 2023, Ji et al., 2024, Fan et al., 2023, Sassi et al., 2015, Yang et al., 2019, Du et al., 2020, and Chi et al., 2024) to visualize sample clustering and assess variance structure within the dataset and confirm clear separations between pure and adulterated milk or different milk origin based on their proteomic profiles [18,91,92,93,126,127,128].

Hierarchical cluster analysis (HCA) complements PCA by grouping samples based on similarity metrics such as Euclidean distance and linkage criteria (e.g., Ward’s method) [129]. This approach is particularly useful for validating sample relationships and identifying subgroups or anomalies within the dataset. Together, PCA and HCA form a complementary toolkit for evaluating the natural structure of the data prior to the application of supervised classification models.

Together, feature selection and exploratory analysis serve as essential precursors to supervised modeling, guiding the refinement of variable sets and offering critical insight into the underlying data structure. These steps ensure that downstream classification and biomarker discovery are based on the most relevant and reproducible features.

### 6.3. Supervised Classification and Quantification Modeling

The implementation of supervised chemometric models in proteomics-based milk authentication is determined by the study objective—whether focused on categorical classification (e.g., milk species identification) or on the quantification of adulterant levels. These two modeling strategies differ in statistical approach, validation outputs, and interpretative metrics.

Supervised classification models are commonly employed to differentiate milk samples of different species or detect adulteration patterns based on proteomic profiles. Among these, partial least squares–discriminant analysis (PLS-DA) has been widely applied in studies utilizing high-dimensional mass spectrometry datasets. For example, Du et al. (2020) used PLS-DA to classify samples based on fused fingerprints of intact and hydrolyzed milk proteins obtained via UPLC-QTOF-MS, while Yang et al. (2019) used it to discriminate between authentic and adulterated milk profiles using spectral protein patterns [126,128].

In studies relying on protein fingerprint, linear discriminant analysis (LDA) has been frequently employed due to its computational efficiency and interpretability. Piras et al. (2020) implemented LDA on AP-MALDI-MS lipid and protein profiles for both milk speciation and adulteration detection, achieving reliable classification even with 10% adulteration [130]. In a subsequent study, Piras et al. (2021) extended the application of LDA to a broader dataset, reporting sensitivity above 92.5% for detecting cow milk in goat/sheep matrices at levels as low as 5% [119]. Similarly, Zhang et al. (2021) used LDA on fluorescence sensor array responses, achieving 100% classification accuracy in identifying cow, goat, camel, and buffalo milk samples [97].

An additional classification strategy was demonstrated by Sassi et al. (2015), who implemented genetic algorithm-based modeling to select discriminative spectral peaks from MALDI-TOF-MS profiles [91]. The modeling procedure involved univariate filtering (Wilcoxon, Anderson–Darling, and *t*-tests with *p* < 1 × 10^−6^), followed by GA-based optimization and internal cross-validation.

In studies where the aim was to estimate the degree of adulteration (typically expressed as the percentage of cow milk present in non-cow milk samples) regression-based models were used. Partial least squares regression (PLSR) was the predominant choice across multiple studies due to its ability to handle multicollinearity and its suitability for high-dimensional datasets. Tehrani et al. (2024) applied PLSR to MALDI-TOF-MS data to quantify cow milk adulteration in goat and sheep milk [16]. Similarly, Du et al. (2020) used PLSR on fused LC-MS/MS fingerprints, while Liu et al. (2023) used fluorescence intensity data to build PLSR models predicting cow milk content in goat milk [128,129]. Sassi et al. (2015) combined PCA and PLSR to predict cow milk adulteration in goat, sheep, and buffalo milk using MALDI-TOF-MS protein profiles [91].

In addition to PLSR, regularized regression models have also been explored. Tehrani et al. (2024) implemented a generalized linear model (GLM) with Lasso regularization to reduce overfitting and improve variable selection in MALDI datasets, an approach particularly useful in sparse high-dimensional feature spaces [16]. Similarly, Piras et al. (2021) utilized GLM-Lasso to predict cow milk percentages in goat and sheep matrices using MS1 peak area data with GLM-Lasso [119]. Zhang et al. (2021) built regression models using LDA-derived factors to predict melamine and soybean milk adulteration, achieving R^2^ > 0.99. Comparable strategies were used in immunoassay-based studies such as that by Ren et al. (2014), which correlated ELISA signal intensity with cow milk concentration [97,99].

### 6.4. Model Validation and Performance Evaluation

The robustness and scientific credibility of any supervised chemometric model, whether designed for classification or regression, depend fundamentally on rigorous validation and transparent performance evaluation. In the context of milk authentication, where predictive outcomes may inform regulatory decisions or support fraud detection, model validation is not optional but essential.

Internal validation, most implemented through cross-validation techniques such as k-fold or repeated sub-sampling, remains the predominant strategy across the literature [122]. For example, Tehrani et al. (2024) applied cross-validation using Haaland’s criterion to evaluate their PLSR models, reporting RMSEP values of 7.7% and 8.8% for cow–goat and cow–sheep quantification, respectively. They also incorporated F-ratio-based outlier detection to ensure model robustness [16]. Similarly, Sassi et al. (2015) relied on the internal validation framework provided by ClinProt, reporting both recognition capability and cross-validation percentages to assess the stability and predictive performance of their GA-based classification models [91].

Piras et al. (2021) employed a particularly robust dual-validation approach, combining internal validation via the built-in “leave 20% out” cross-validation function of the AMX (abstract model builder) with external validation. Their classification and quantification models were tested on an independent test set representing one-third of their full dataset [119].

External validation—though less frequently performed due to sample size constraints—offers the most rigorous assessment of a model’s generalizability. Liu et al. (2023) implemented a clear train/test data split, reserving 81 samples for model validation [129]. Their PLSR model achieved calibration and prediction R^2^ values of 0.932 and 0.951, respectively, with RMSECV and RMSEP values of 0.676% and 0.457%, indicating high predictive accuracy and stability.

In contrast, some high-performing models—such as those described by Zhang et al. (2021)—reported exceptionally high internal calibration statistics (e.g., R^2^ > 0.99) yet lacked formal external validation or permutation testing [97]. This highlights a broader challenge in the field: while internal validation is necessary, it is insufficient on its own to guarantee real-world applicability.

In such cases, caution is warranted, as extremely high R^2^ values may indicate potential overfitting—particularly when no external or independent validation is performed. Future studies should prioritize the use of external datasets or robust cross-validation frameworks to ensure model generalizability and reproducibility.

### 6.5. Data Fusion Strategies

Data fusion offers a powerful approach to enhance the performance of chemometric models by integrating complementary information from multiple data sources. Three major fusion architectures are generally recognized: low-level fusion, which involves direct concatenation of features; mid-level fusion, where latent variables or selected features are merged; and high-level fusion, in which model outputs are combined for decision-level integration [131].

In milk authentication, the most notable application comes from Du et al. (2020), who employed both low- and mid-level fusion strategies. In their study, low-level fusion was performed by directly concatenating the full feature matrices from the two datasets. PCA on the fused matrix improved the separation of authentic and adulterated milk compared to PCA on individual datasets. To further enhance model performance, mid-level fusion was implemented by extracting latent variables from separate PLS-DA models applied to each dataset. The resulting PLS-DA scores were merged and subjected to PCA, yielding complete discrimination between authentic and adulterated samples—even at 0.5% adulteration. The authors noted that mid-level fusion reduced dimensionality while retaining key discriminatory features, and it outperformed the low-level approach [128].

Nevertheless, as multi-source and multi-omics datasets become more common, data fusion represents a promising area for future development in milk authentication research.

## 7. Conclusions and Future Directions

Proteins and peptides have emerged as promising biomarkers for the authentication of milk and dairy products, each with unique strengths and limitations.

Proteins offer high specificity as biomarkers due to their well-defined primary, secondary, and tertiary structures, providing unique profiles for differentiating animal species. Their detailed characterization through advanced analytical techniques ensures robust and reliable authentication. However, proteins are prone to degradation during food processing [132], storage, and handling, which limits their applicability in processed dairy products. Furthermore, structural and functional alterations caused by genetic variations [133] can reduce their reliability as biomarkers.

Peptides, on the other hand, exhibit greater stability under harsh processing conditions such as high temperatures, pH modifications, and ionic strength changes [134]. This makes them particularly suitable for processed dairy products where intact proteins may degrade. Nevertheless, peptide formation during processing can also be influenced by these treatments. Their smaller size facilitates deeper insights into proteolytic processes and species-specific modifications. While many peptides have shown promising species specificity, particularly in bottom-up proteomics workflows, their discriminatory power can be limited when sequences are conserved across related species. Additionally, their identification requires time-intensive sample preparation with enzymatic digestion, such as trypsin hydrolysis [66,112,114,115,116,117].

To enhance the reliability and applicability of protein and peptide biomarkers for milk and dairy product authentication, the following areas require further exploration:

- **Geographical Origin Profiling**: Differentiating protein and peptide profiles based on geographical origin remains underexplored. While environmental factors including feed composition, climate, and farming practices are believed to influence protein and peptide profiles, systematic studies are lacking. Investigating variations of the same protein or peptide within the same animal species across different regions could identify subtle, consistent geographical markers. Such studies are essential for combating fraud and protecting products with geographical designations such as PDO and protected geographical indication (PGI) while promoting fair trade practices;
- **Effect of Breed on Protein Profiles**: The influence of animal breeds on milk protein and peptide profiles remains understudied. Detailed comparisons between breeds can provide valuable insights into intra-species variations, improving species differentiation and quality assessments. Such studies would establish comprehensive protein and peptide reference databases [135,136,137];
- **Data Reliability and Sampling Protocols**: Reliable biomarker development requires standardized methodologies for sample collection, storage, and analysis. Future studies should adopt longitudinal sampling approaches that account for lactation stages, seasonal variations, and the physiological states of animals [138,139,140]. Ensuring comprehensive and high-quality data will strengthen the reproducibility and validity of biomarker-based authentication methods;
- **Integrative Multi-Omics and Data Fusion Strategies:** The future of milk authentication lies in the integration of complementary omics technologies—such as proteomics, metabolomics, genomics, and isotopic profiling—within unified analytical frameworks [141]. While each omics platform provides distinct insights, their combination through structured data fusion approaches can substantially improve the sensitivity, specificity, and robustness of authenticity assessments. The integration of proteomics and metabolomics through structured data fusion has proven essential for advancing foodomics, particularly in complex matrices like milk. Complementing this, Blanchet and Smolinska (2016) proposed a two-step fusion framework where each omics dataset is first compressed to extract the most relevant variables and subsequently merged to allow joint analysis [131]. Together, these strategies enable a more holistic understanding of molecular mechanisms;
- **Machine learning (ML):** Recent advances in machine learning (ML)-assisted proteomics offer promising avenues to strengthen milk authentication workflows. Li et al. (2025) emphasized that deep learning models such as convolutional neural network (CNN)s and residual networks can enhance peptide identification and retention time prediction. However, challenges remain, including limited dataset diversity, insufficient multimodal integration, and the absence of standardized validation protocols. Future research should focus on developing robust, large-scale datasets and combining data-driven and mechanistic modeling approaches to improve accuracy, interpretability, and reproducibility [142].

Despite their proven analytical power, protein- and peptide-based authentication methods, particularly those involving mass spectrometry, face practical limitations in real-world settings. These include high equipment and maintenance costs, limited accessibility in small or regional laboratories, the need for highly trained personnel, and challenges in cross-platform standardization. As a result, there is a growing need for simplified, cost-effective protocols and user-friendly computational tools that can facilitate wider adoption in regulatory, industrial, and low-resource environments.

By addressing these critical areas, the scientific community can refine and expand the application of protein and peptide biomarkers. A multidisciplinary approach combining advanced proteomic analytical tools, bioinformatics, and standardized methodologies will ensure the development of robust, reliable, and comprehensive authentication techniques for milk. These efforts will contribute significantly to combatting food fraud, protecting geographical designations, and ensuring consumer confidence in dairy products.

## Figures and Tables

**Table 1:** Key Analytical Platforms in Milk Authentication: Strengths and Limitations.

Technique	Advantages	Limitations
Electrophoretic techniques (SDS-PAGE/IEF/2D-PAGE) [44,45]	Cost effective; useful for initial protein separation; can resolve isoforms	Low specificity; overlapping bands; limited in processed samples; requires standards for IEF
Immunoassays (e.g., ELISA) [25,50]	High sensitivity and specificity; fast; suitable for routine screening	Cross-reactivity; ineffective if target proteins are degraded
Mass Spectrometry-Based Proteomics (LC-MS/MS, HPLC-QTOF, and MALDI-TOF) [51,52,53,54,55,56,57,58,59,60,61].	High multiplexing and quantification capability; species-level resolution	High cost; requires advanced instruments, skilled personnel, and complex data processing
Spectroscopic Methods (e.g., FTIR, NIR, and NMR)	Rapid; non-destructive; high throughput; some methods are portable	Dependent on large spectral databases; expensive for high-resolution platforms
DNA-Based Methods (PCR, qPCR, and RFLP) [66,67]	High specificity; good for detecting species substitution	Not suited for quantification; affected by DNA degradation in processed milk
Isotopic and Elemental Fingerprinting [70,71]	Accurate for geographical origin authentication	High equipment cost; may require large datasets; indirect biological relevance
Biosensors [69]	Low cost; fast; portable; user-friendly	Often qualitative; sensitivity limitations; limited multiplexing

**Table 2:** Protein content (%) in the milk of some species from Fox et al. (2015) [81].

Species	Casein	Whey Proteins	Total
Buffalo	3.5–4.2	0.92	4.42–5.12
Camel (bactrian)	2.9	1.0	3.9
Cow	2.8	0.6	3.4
Donkey	1.0	1.0	2.0
Goat	2.5	0.4	2.9
Horse	1.3	1.2	2.54

**Table 3:** Animal species, amino acid total number, and molecular weights of caseins in five dairy animals [118].

Casein (Gene)	Animal S.N. (En.) Names	Total Number of Amino Acids	M.W. kDa
*α-S1-casein (CSN1S1)*	*Camelus dromedaries* (Arabian camels)	222	25.843
	*Ovis aries* (sheep)	214	24.315
	*Capra hircus* (goats)	213	24.13
	*Bos taurus* (cattle)	214	24.435
	*Bubalus bubalis* (water buffalos)	214	24.312
*α-S2-casein (CSN1S2)*	*Camelus dromedaries* (Arabian camels)	193	22.964
	*Ovis aries* (sheep)	223	26.331
	*Capra hircus* (goats)	223	26.341
	*Bos taurus* (cattle)	222	26.12
	*Bubalus bubalis* (water buffalos)	222	26.223
*β-casein (CSN2)*	*Camelus dromedaries* (Arabian camels)	232	26.217
	*Ovis aries* (sheep)	222	24.946
	*Capra hircus* (goats)	223	24.992
	*Bos taurus* (cattle)	223	25.098
	*Bubalus bubalis* (water buffalos)	224	25.101
*κ-casein (CSN3)*	*Camelus dromedaries* (Arabian camels)	219	24.717
	*Ovis aries* (sheep)	162	17.899
	*Capra hircus* (goats)	162	17.896
	*Bos taurus* (cattle)	190	21.269
	*Bubalus bubalis* (water buffalos)	190	21.397

**Table 4:** Peptide Biomarkers for Milk Authentication using Advanced Analytical Techniques.

Marker Proteins	Marker Peptide Sequence	Milk Origin	Aim of the Study	Analytical Technique	Detection Limit	Ref.
**αs_1_-casein**	FFVAPFPEVFGK (cow)	Camel	Analyze major camel and cow milk proteins through selected stable marker peptides and detect adulteration with cow milk	Identification of digested peptides: UPLC-ESI-TOF-MS (+); Quantitative analysis of peptides: UPLC-ESI-QQQMS (MRM)	0.101 ng/mL	[114]
	FFVAPFPEVFGK (38–49) (cow)	Cow, Goat, Sheep	Quantify cow’s whey and whole-milk powder percentage in goat or sheep milk products, including infant formula	UHPLC-ESI-TOF-MS (+)	0.01–0.05 g/100 g cow’s whey and whole-milk powder in goat’s or sheep’s milk products, including infant formula	[115]
	FVVAPFPEVFR (38–48) (Goat and Sheep)	Cow, Goat, Sheep	Quantify cow’s whey and whole-milk powder percentage in goat or sheep milk products, including infant formula	UHPLC-ESI-TOF-MS (+)	0.01–0.05 g/100 g cow’s whey and whole-milk powder in goat’s or sheep’s milk products, including infant formula	[115]
	VNELSK (52–57) (cow)	Cow, Goat, Sheep	Quantify cow’s whey and whole-milk powder percentage in goat or sheep milk products, including infant formula	UHPLC-ESI-TOF-MS (+)	0.01–0.05 g/100 g cow’s whey and whole-milk powder in goat’s or sheep’s milk products, including infant formula	[115]
	ENINELSK (50–57) (Goat and Sheep)	Cow, Goat, Sheep	Quantify cow’s whey and whole-milk powder percentage in goat or sheep milk products, including infant formula	UHPLC-ESI-TOF-MS (+)	0.01–0.05 g/100 g cow’s whey and whole-milk powder in goat’s or sheep’s milk products, including infant formula	[66]
	EEYINELNR (Donkey)	Camel, Donkey, Goat, Sheep, Yak, Cow	Identify specific peptide markers of seven milk species and assess the impact of processing treatments for accurate quantification of cow milk adulteration in non-cow milk samples.	HPLC-QTOF-MS (DIA)	1% cow’s milk	[112]
	HQGLPQEVLNENLLR (cow)	Camel, Cow, Water Buffalo, Donkey, Goat, Horse, Sheep	Use signature peptides to measure αs2-caseins, β-caseins, and κ-caseins from eight milk species, enabling accurate detection and evaluation of milk adulteration.	UHPLC-ESI-Orbitrap-MS (+)	LOD: 5 μg/L	[66]
	HQGLPQEVLNENLLR (1759.9449 *m*/*z*) (cow)	Cow, Goat	Inspect adulteration in goat milk, characteristic peptides of caseins from cow milk were screened out	MALDI-TOF/TOF (+)	1% cow’s milk in goat milk	[117]
	αs_1_-casein HQGLPQEVLNENLLR (8–22) (cow)	Goat	Detect cow milk contamination in goat milk	nanoLC-ESI-IT-MS/MS (+) DDA	1% of cow’s milk in goat milk	[116]
	YNQLQLQAIYAQEQLIR (Donkey)	Buffalo, Cow, Donkey, Goat, Sheep, Yak	Identify specific peptide markers of seven milk species and assess the impact of processing treatments for accurate quantification of cow milk adulteration in non-cow milk samples	HPLC-QTOF-MS (DIA)	1% cow’s milk	[112]
	YNQLQLQAIYAQEQLIR (Donkey)	Cow, Water Buffalo, Yak, Goat, sheep, donkey, horse, camel	Use signature peptides to measure αs2-caseins, β-caseins, and κ-caseins from eight milk species, enabling accurate detection and evaluation of milk adulteration	UHPLC-ESI-Orbitrap-MS (+)	LOD: 10 μg/L; LOQ: 20 μg/L	[66]
**as_2_-casein**	NMAINPSK (Cow)	Camel, Donkey, Goat, Sheep, Yak, Cow	Identify specific peptide markers of seven milk species and assess the impact of processing treatments for accurate quantification of cow milk adulteration in non-cow milk samples	HPLC-QTOF-MS (DIA)	1% cow’s milk	[112]
	NMAIHPSK (Buffalo)	Camel, Donkey, Goat, Sheep, Yak, Cow	Identify specific peptide markers of seven milk species and assess the impact of processing treatments for accurate quantification of cow milk adulteration in non-cow milk samples	HPLC-QTOF-MS (DIA)	1% cow’s milk	[112]
	NHLNFLK (Sheep)	Camel, Donkey, Goat, Sheep, Yak, Cow	Identify specific peptide markers of seven milk species and assess the impact of processing treatments for accurate quantification of cow milk adulteration in non-cow milk samples	HPLC-QTOF-MS (DIA)	1% cow’s milk	[112]
	IVLTPWDQTK (Donkey)	Camel, Donkey, Goat, Sheep, Yak, Cow	Identify specific peptide markers of seven milk species and assess the impact of processing treatments for accurate quantification of cow milk adulteration in non-cow milk samples	HPLC-QTOF-MS (DIA)	1% cow’s milk	[112]
	TNSYQIIPVLR (Donkey)	Camel, Donkey, Goat, Sheep, Yak, Cow	Identify specific peptide markers of seven milk species and assess the impact of processing treatments for accurate quantification of cow milk adulteration in non-cow milk samples	HPLC-QTOF-MS (DIA)	1% cow’s milk	[112]
	LNFLQYLQALR (Donkey)	Camel, Donkey, Goat, Sheep, Yak, Cow	Identify specific peptide markers of seven milk species and assess the impact of processing treatments for accurate quantification of cow milk adulteration in non-cow milk samples	HPLC-QTOF-MS (DIA)	1% cow’s milk	[112]
	ISQHYQK (Buffalo)	Camel, Donkey, Goat, Sheep, Yak, Cow	Identify specific peptide markers of seven milk species and assess the impact of processing treatments for accurate quantification of cow milk adulteration in non-cow milk samples	HPLC-QTOF-MS (DIA)	1% cow’s milk	[112]
	TNVIPYVR (Buffalo)	Camel, Donkey, Goat, Sheep, Yak, Cow	Identify specific peptide markers of seven milk species and assess the impact of processing treatments for accurate quantification of cow milk adulteration in non-cow milk samples	HPLC-QTOF-MS (DIA)	1% cow’s milk	[112]
	AMKPWIQPK (Cow)	Camel, Donkey, Goat, Sheep, Yak, Cow	Identify specific peptide markers of seven milk species and assess the impact of processing treatments for accurate quantification of cow milk adulteration in non-cow milk samples	HPLC-QTOF-MS (DIA)	1% cow’s milk	[112]
	LCTTSCEEVVR (51–61) (Goat and Sheep)	Cow, Goat, Sheep	Quantify cow’s whey and whole-milk powder percentage in goat or sheep milk products, including infant formula	UHPLC-ESI-TOF-MS (+)	0.01–0.05 g/100 g cow’s whey and whole-milk powder in goat’s or sheep’s milk products, including infant formula	[115]
	NAVPITPTLNR (1195.6793 *m*/*z*) (cow)	Cow, Goat	Inspect adulteration in goat milk; screen out characteristic peptides of caseins from cow milk	MALDI-TOF/TOF (+)	1% cow’s milk in goat milk	[117]
	NAVPITPTLNR (131–141) (cow)	Cow, Goat, Sheep	Quantify cow’s whey and whole-milk powder percentage in goat or sheep milk products, including infant formula	UHPLC-ESI-TOF-MS (+)	0.01–0.05 g/100 g cow’s whey and whole-milk powder in goat’s or sheep’s milk products, including infant formula	[115]
	NAGPFTPTVNR (131–141) (Sheep and Goat)	Cow, Goat, Sheep	Quantify cow’s whey and whole-milk powder percentage in goat or sheep milk products, including infant formula	UHPLC-ESI-TOF-MS (+)	0.01–0.05 g/100 g cow’s whey and whole-milk powder in goat’s or sheep’s milk products, including infant formula	[115]
	ENLCSTFCK (49–57) (cow)	Cow, Goat, Sheep	Quantification of cow’s whey and whole-milk powder percentage in goat or sheep milk products, including infant formula	UHPLC-ESI-TOF-MS (+)	0.01–0.05 g/100 g cow’s whey and whole-milk powder in goat’s or sheep’s milk products, including infant formula	[115]
	TVYQHQK (198–204) (cow)	Cow, Goat, Sheep	Quantification of cow’s whey and whole-milk powder percentage in goat or sheep milk products, including infant formula	UHPLC-ESI-TOF-MS (+)	0.01–0.05 g/100 g cow’s whey and whole-milk powder in goat’s or sheep’s milk products, including infant formula	[115]
	TVYQHQK (198–204) (Goat and Sheep)	Cow, Goat, Sheep	Quantification of cow’s whey and whole-milk powder percentage in goat or sheep milk products, including infant formula	UHPLC-ESI-TOF-MS (+)	0.01–0.05 g/100 g cow’s whey and whole-milk powder in goat’s or sheep’s milk products, including infant formula	[115]
	FALPQYLK (cow)	Camel	Develop and validate a method using mass spectrometry to quantitatively analyze major camel and cow milk proteins through selected stable marker peptides and detect adulteration with cow milk	Identification of digested peptides: UPLC-ESI-TOF-MS (+); Quantitative analysis of peptides: UPLC-ESI-TQMS (MRM)	0.045 ng/mL	[114]
	FALPQYLK (190–197) (cow)	Cow, Goat, Sheep	Quantify cow’s whey and whole-milk powder percentage in goat or sheep milk products, including infant formula	UHPLC-ESI-TOF-MS (+)	0.01–0.05 g/100 g cow’s whey and whole-milk powder in goat’s or sheep’s milk products including infant formula	[115]
	FALPQYLK (190–197) (Goat and Sheep)	Cow, Goat, Sheep	Quantify cow’s whey and whole-milk powder percentage in goat or sheep milk products, including infant formula	UHPLC-ESI-TOF-MS (+)	0.01–0.05 g/100 g cow’s whey and whole-milk powder in goat’s or sheep’s milk products, including infant formula	[115]
	FPQYLQYPYQGPIVLNPWDQVK (Goat)	Camel, donkey, Yak, goat, cow, sheep	Identify specific peptide markers of seven milk species and assess the impact of processing treatments for accurate quantification of cow milk adulteration in non-cow milk samples	HPLC-QTOF-MS (DIA)	1% cow’s milk	[112]
	VLPVPQK (Cow)	Camel	Develop and validate a method using mass spectrometry to quantitatively analyze major camel and cow milk proteins through selected stable marker peptides and detect adulteration with cow milk	Identification of digested peptides: UPLC-ESI-TOF-MS (+;) Quantitative analysis of peptides: UPLC-ESI-TQMS (MRM)	0.004 ng/mL	[114]
**β-casein**	AVPYPQR (830.4519 *m*/*z*) (Cow)	Cow, Goat	Inspect adulteration in goat milk; screen out characteristic peptides of caseins from cow milk	MALDI-TOF/TOF (+)	1% cow’s milk in goat milk	[117]
	YPVEPFTER (Cow)	Camel, Donkey, Goat, Sheep, Yak, Cow	Identify specific peptide markers of seven milk species and assess the impact of processing treatments for accurate quantification of cow milk adulteration in non-cow milk samples	HPLC-QTOF-MS (DIA)	1% cow’s milk	[112]
	IEEQQQTEDEQQDK (Camel)	Camel, Donkey, Goat, Sheep, Yak, Cow	Identify specific peptide markers of seven milk species and assess the impact of processing treatments for accurate quantification of cow milk adulteration in non-cow milk samples	HPLC-QTOF-MS (DIA)	1% cow’s milk	[112]
	FQSEEQQQMEDELQDK (Buffalo)	Camel, Donkey, Goat, Sheep, Yak, Cow	Identify specific peptide markers of seven milk species and assess the impact of processing treatments for accurate quantification of cow milk adulteration in non-cow milk samples	HPLC-QTOF-MS (DIA)	1% cow’s milk	[112]
	IHPFAQTQSLVYPFPGPIPK (Buffalo)	Camel, Donkey, Goat, Sheep, Yak, Cow	Identify specific peptide markers of seven milk species and assess the impact of processing treatments for accurate quantification of cow milk adulteration in non-cow milk samples	HPLC-QTOF-MS (DIA)	1% cow’s milk	[112]
	AIPVQAVLPFQEPVPDPVR (Camel)	Camel, Donkey, Goat, Sheep, Yak, Cow	Identify specific peptide markers of seven milk species and assess the impact of processing treatments for accurate quantification of cow milk adulteration in non-cow milk samples	HPLC-QTOF-MS (DIA)	1% cow’s milk	[112]
	VAPFPQPVVPYPQR (Donkey)	Camel, Donkey, Goat, Sheep, Yak, Cow	Identify specific peptide markers of seven milk species and assess the impact of processing treatments for accurate quantification of cow milk adulteration in non-cow milk samples	HPLC-QTOF-MS (DIA)	1% cow’s milk	[112]
	GPFPIIV (217–224) (Goat and Sheep)	Cow, Goat, Sheep	Quantify cow’s whey and whole-milk powder percentage in goat or sheep milk products, including infant formula	UHPLC-ESI-TOF-MS (+)	0.01–0.05 g/100 g cow’s whey and whole-milk powder in goat’s or sheep’s milk products, including infant formula	[115]
	YIPIQYVLSR	Camel	Develop and validate a method using mass spectrometry to quantitatively analyze major camel and cow milk proteins through selected stable marker peptides and detect adulteration with cow milk	Identification of digested peptides: UPLC-ESI-TOF-MS (+); Quantitative analysis of peptides: UPLC-ESI-TQMS (MRM)	0.103 ng/mL	[114]
**κ-casein**	FFSDK (38–42) (Cow)	Cow, Goat, Sheep	Quantify cow’s whey and whole-milk powder percentage in goat or sheep milk products, including infant formula	UHPLC-ESI-TOF-MS (+)	0.01–0.05 g/100 g cow’s whey and whole-milk powder in goat’s or sheep’s milk products, including infant formula	[115]
	FFDDK (38–42) (Goat and Sheep)	Cow, Goat, Sheep	Quantify cow’s whey and whole-milk powder percentage in goat or sheep milk products including infant formula	UHPLC-ESI-TOF-MS (+)	0.01–0.05 g/100 g cow’s whey and whole-milk powder in goat’s or sheep’s milk products, including infant formula	[115]
	SPAQTLQWQVLPNTVPAK (Goat)	Camel, Donkey, Goat, Sheep, Yak, Cow	Identify specific peptide markers of seven milk species and assess the impact of processing treatments for accurate quantification of cow milk adulteration in non-cow milk samples	HPLC-QTOF-MS (DIA)	1% cow’s milk	[112]
	SPAQTLQWQVLPNTVPAK (Goat)	Cow, Water Buffalo, Yak, Goat, sheep, donkey, horse, camel	Use signature peptides to measure αs2-caseins, β-caseins, and κ-caseins from eight milk species, enabling accurate detection and evaluation of milk adulteration	UHPLC-ESI-Orbitrap-MS (+)	LOD: 10 μg/L; LOQ: 30 μg/L	[66]
	SPAQTLQWQVLPNAVPAK (Sheep)	Camel, Donkey, Goat, Sheep, Yak, Cow	Identify specific peptide markers of seven milk species and assess the impact of processing treatments for accurate quantification of cow milk adulteration in non-cow milk samples	HPLC-QTOF-MS (DIA)	1% cow’s milk	[112]
	SPAQTLQWQVLPNAVPAK (Sheep)	Cow, Water Buffalo, Yak, Goat, sheep, donkey, horse, camel	Use signature peptides to measure αs2-caseins, β-caseins, and κ-caseins from eight milk species, enabling accurate detection and evaluation of milk adulteration	UHPLC-ESI-Orbitrap-MS (+)	LOD: 10 μg/L; LOQ: 30 μg/L	[3]
	SPAQILQWQVLPNTVPAK (Buffalo)	Camel, Donkey, Goat, Sheep, Yak, Cow	Identify specific peptide markers of seven milk species and assess the impact of processing treatments for accurate quantification of cow milk adulteration in non-cow milk samples	HPLC-QTOF-MS (DIA)	1% cow’s milk	[112]
	SCQAQPTTMAR (Cow)	Camel, Donkey, Goat, Sheep, Yak, Cow	Identify specific peptide markers of seven milk species and assess the impact of processing treatments for accurate quantification of cow milk adulteration in non-cow milk samples	HPLC-QTOF-MS (DIA)	1% cow’s milk	[112]
	SCQDQPTAMAR (Sheep)	Camel, Donkey, Goat, Sheep, Yak, Cow	Identify specific peptide markers of seven milk species and assess the impact of processing treatments for accurate quantification of cow milk adulteration in non-cow milk samples	HPLC-QTOF-MS (DIA)	1% cow’s milk	[112]
	YFPIQFVQSR (Camel)	Camel, Donkey, Goat, Sheep, Yak, Cow	Identify specific peptide markers of seven milk species and assess the impact of processing treatments for accurate quantification of cow milk adulteration in non-cow milk samples	HPLC-QTOF-MS (DIA)	1% cow’s milk	[112]
	IDALNENK (Cow)	Camel	Analyze major camel and cow milk proteins through selected stable marker peptides and detect adulteration with cow milk	Identification of digested peptides: UPLC-ESI-TOF-MS (+); Quantitative analysis of peptides: UPLC-ESI-TQMS (MRM)	0.066 ng/mL	[114]
	LSFNPTQLEEQCHI (167–180) (cow)	Cow, Goat, Sheep	Quantify cow’s whey and whole-milk powder percentage in goat or sheep milk products, including infant formula	UHPLC-ESI-TOF-MS (+)	0.01–0.05 g/100 g cow’s whey and whole-milk powder in goat’s or sheep’s milk products, including infant formula	[115]
	β-Lactoglobulin LSFNPTQLEEQCHI (149–162)	Goat	Detect cow milk contamination in goat milk	nanoLC-ESI-IT-MS/MS (+) DDA	1% of cow’s milk in goat milk	[116]
**β-lactoglobulin**	LAFNPTQLEGQCHV (167–180) (Goat and Sheep)	Cow, Goat, Sheep	Quantify cow’s whey and whole-milk powder percentage in goat or sheep milk products, including infant formula	UHPLC-ESI-TOF-MS (+)	0.01–0.05 g/100 g cow’s whey and whole-milk powder in goat’s or sheep’s milk products, including infant formula	[115]
	LSFNPTQLEGQCHI (Yak)	Camel, Donkey, Goat, Sheep, Yak, Cow	Identify specific peptide markers of seven milk species and assess the impact of processing treatments for accurate quantification of cow milk adulteration in non-cow milk samples	HPLC-QTOF-MS (DIA)	1% cow’s milk	[112]
	WENDECAQK (Cow)	Camel, Donkey, Goat, Sheep, Yak, Cow	Identify specific peptide markers of seven milk species and assess the impact of processing treatments for accurate quantification of cow milk adulteration in non-cow milk samples	HPLC-QTOF-MS (DIA)	1% cow’s milk	[112]
	LSFNPTQLEGQCHI (Yak)	Camel, Donkey, Goat, Sheep, Yak, Cow	Identify specific peptide markers of seven milk species and assess the impact of processing treatments for accurate quantification of cow milk adulteration in non-cow milk samples	HPLC-QTOF-MS (DIA)	1% cow’s milk	[112]
	NICNISCDK (90–98) (Goat and Sheep)	Cow, Goat, Sheep	Quantify cow’s whey and whole-milk powder percentage in goat or sheep milk products, including infant formula	UHPLC-ESI-TOF-MS (+)	0.01–0.05 g/100 g cow’s whey and whole-milk powder in goat’s or sheep’s milk products, including infant formula	[115]
	CEVFR (25–29) (Cow)	Cow, Goat, Sheep	Quantify cow’s whey and whole-milk powder percentage in goat or sheep milk products, including infant formula	UHPLC-ESI-TOF-MS (+)	0.01–0.05 g/100 g cow’s whey and whole-milk powder in goat’s or sheep’s milk products, including infant formula	[115]
**α-lactalbumin**	NICNISCDK (90–98) (Goat and Sheep)	Cow, Goat, Sheep	Quantify cow’s whey and whole-milk powder percentage in goat or sheep milk products, including infant formula	UHPLC-ESI-TOF-MS (+)	0.01–0.05 g/100 g cow’s whey and whole-milk powder in goat’s or sheep’s milk products, including infant formula	[115]
	CEVFR (25–29) (Cow)	Cow, Goat, Sheep	Quantify cow’s whey and whole-milk powder percentage in goat or sheep milk products, including infant formula	UHPLC-ESI-TOF-MS (+)	0.01–0.05 g/100 g cow’s whey and whole-milk powder in goat’s or sheep’s milk products, including infant formula	[115]

**Table 5:** Index of Peptide Biomarkers for Milk of Table 4.

Marker Proteins	Marker Peptide Sequence	Milk Origin	Ref.
**αs_1_-casein**	FFVAPFPEVFGK (38–49)	Cow	[114,115]
	FVVAPFPEVFR (38–48)	Goat and Sheep	[115]
	VNELSK (52–57)	Cow	[115]
	ENINELSK (50–57)	Goat and Sheep	[66]
	EEYINELNR	Donkey	[112]
	HQGLPQEVLNENLLR (1759.9449 *m*/*z*)	Cow	[117]
	YNQLQLQAIYAQEQLIR	Donkey	[66,112]
**as_2_-casein**	NMAINPSK	Cow	[112]
	NMAIHPSK	Buffalo	[112]
	NHLNFLK	Sheep	[112]
	IVLTPWDQTK	Donkey	[112]
	TNSYQIIPVLR	Donkey	[112]
	LNFLQYLQALR	Donkey	[112]
	ISQHYQK	Buffalo	[112]
	TNVIPYVR	Buffalo	[112]
	AMKPWIQPK	Cow	[112]
	LCTTSCEEVVR (51–61)	Goat and Sheep	[115]
	NAVPITPTLNR (131–141) (1195.6793 *m*/*z*)	Cow	[115,117]
	NAGPFTPTVNR (131–141)	Sheep and Goat	[115]
	ENLCSTFCK (49–57)	Cow	[115]
	TVYQHQK (198–204)	Cow	[115]
	FALPQYLK (190–197)	Cow	[114,115]
	FPQYLQYPYQGPIVLNPWDQVK	Goat	[112]
	VLPVPQK	Cow	[114]
**β-casein**	AVPYPQR (830.4519 *m*/*z*)	Cow	[117]
	YPVEPFTER	Cow	[112]
	IEEQQQTEDEQQDK	Camel	[112]
	FQSEEQQQMEDELQDK	Buffalo	[112]
	IHPFAQTQSLVYPFPGPIPK	Buffalo	[112]
	AIPVQAVLPFQEPVPDPVR	Camel	[112]
	VAPFPQPVVPYPQR	Donkey	[112]
	GPFPIIV (217–224)	Goat and Sheep	[115]
	YIPIQYVLSR	Cow	[114]
**κ-casein**	FFSDK (38–42)	Cow	[115]
	SPAQTLQWQVLPNTVPAK	Goat	[66,112]
	SPAQTLQWQVLPNAVPAK	Sheep	[112]
	SPAQILQWQVLPNTVPAK	Buffalo	[112]
	SCQAQPTTMAR	Cow	[112]
	SCQDQPTAMAR	Sheep	[112]
	YFPIQFVQSR	Camel	[112]
	IDALNENK	Cow	[114]
	LSFNPTQLEEQCHI (167–180)	Cow	[115]
**β-lactoglobulin**	LAFNPTQLEGQCHV (167–180)	Goat and Sheep	[115]
	LSFNPTQLEGQCHI	Yak	[112]
	WENDECAQK	Cow	[112]
**α-lactalbumin**	NICNISCDK (90–98)	Goat and Sheep	[115]
	CEVFR (25–29)	Cow	[115]

## Supplementary Materials

The following supporting information can be downloaded at https://www.mdpi.com/article/10.3390/foods14152588/s1, Table S1: Protein Biomarkers for Milk Authentication using Advanced Analytical Techniques.

## Abbreviations

The following abbreviations are used in this manuscript:

AP-MALDI	Atmospheric Pressure–Matrix-Assisted Laser Desorption/Ionization
BSA	Bovine Serum Albumin
CE	Capillary Electrophoresis
cGMP	Casein Glycomacropeptide
CMP	Caseinomacropeptide
CN	Casein
DDA	Data-Dependent Acquisition
DIA	Data-Independent Acquisition
ELISA	Enzyme-Linked Immunosorbent Assay
EMA	Economically Motivated Adulteration
ESI-MS	Electrospray Ionization–Mass Spectrometry
EU	European Union
FAO	Food and Agriculture Organization of the United Nations
FDA	Food and Drug Administration
FTIR	Fourier-Transform Infrared Spectroscopy
GLM-Lasso	Generalized Linear Model with Lasso regularization
GMP	Glycomacropeptide
IEF	Isoelectric Focusing
IgG	Immunoglobulin G
LC-MS/MS	Liquid Chromatography–Mass Spectrometry
LDA	Linear Discriminant Analysis
LFIA	Lateral Flow Immunoassay
MALDI-TOF-MS	Matrix-Assisted Laser Desorption/Ionization–Time-of-Flight–Mass Spectrometry
MFGM	Milk Fat Globule Membrane
MID	Mid-Infrared Spectroscopy
MS	Mass Spectrometry
MMP	Mare Milk Powder
NIR	Near-Infrared Spectroscopy
nLC-MS/MS	Nano Liquid Chromatography–Mass Spectrometry
NLISA	Nanozyme-Linked Immunosorbent Assay
NMR	Nuclear Magnetic Resonance
OECD	Organization for Economic Co-operation and Development
P	Phosphorylation
PAGIF	Polyacrylamide Gel
PCA	Principal Component Analysis
PCR	Polymerase Chain Reaction
PDO	Protected Designation of Origin
PGI	Protected Geographical Indication
PMM	Pasteurized Mare Milk
PLS-DA	Partial Least Squares–Discriminant Analysis
PRISMA	Preferred Reporting Items for Systematic Reviews and Meta-Analyses
PTM	Post-Translational Modification
QCM	Quartz Crystal Microbalance
RSD	Relative Standard Deviation
SDS-PAGE	Sodium Dodecyl Sulfate Polyacrylamide Gel Electrophoresis
TSG	Traditional Specialty Guaranteed
UHPLC-ESI-QTOF-MS	Ultra-High Performance Liquid Chromatography–Electrospray Ionization–Quadrupole Time-of-Flight–Mass Spectrometry
UPLC-DAD	Ultra-Performance Liquid Chromatography–Diode Array Detector
α-La	Alpha-Lactalbumin
αs1-CN	Alpha-s1 Casein
αs2-CN	Alpha-s2 Casein
β-CN	Beta Casein
β-lg	Beta-Lactoglobulin
γ-CN	Gamma Casein
κ-CN	Kappa Casein
