# Supplementary material for: Assessing Milk Authenticity Using Protein and Peptide Biomarkers: A Decade of Progress in Species Differentiation and Fraud Detection
Source: Foods. 2025 Jul 23;14(15):2588. doi: 10.3390/foods14152588 (PMC12346290; doi:10.3390/foods14152588)
Supplement: Supplementary file 1 [file foods-14-02588-s001.zip › foods-3736849-supplementary.pdf]

Table S1. Protein Biomarkers for Milk Authentication using Advanced Analytical Techniques.

Protein Categories	Marker proteins	Aim of the study	Milk Origin	Analytical tecnique	Detection limit	Ref.
	cow milk casein	Measure the percentage of cow's whole milk contamination or adulteration in goat milk	Goat	Competitive Lateral Flow immunoassay (LFIA) strip	0.07% cow's milk in goat milk	[140]
	α-casein	Detecting milk adulteration using cheese whey, compared to SDS-PAGE	Cow	Microfluidic chip electrophoresis	1% whey in milk (α-casein)	[95]
	α-casein	Detects adulteration with soybean milk in cow milk	Cow	Fluorescence Spectroscopy	0.1% soybean's milk in cow milk	[97]
	αs–casein fractions (peak Ee)	Detection of sheep milk adulteration with cow milk	Sheep	Capillary electrophoresis (CE)	5% of cow's milk in sheep milk	[96]
	αs_1-casein	Protein profile from local Pakistani milk breeds animals such as Nilli-Ravi buffalo, Sahiwal cow, Kajli sheep, Beetal goat and Brela camel	Buffalo (Nilli-Ravi), Cow (Sahiwal), Sheep (Kajli), Goat (Beetal)	SDS-PAGE	Not determined	[98]
	αs_1-casein (cow)	Detection of cow milk in donkey milk	Donkey	1. Isoelectric focusing (IEF) on polyacrylamide gel (PAGIF)	5% (v/v) of cow's milk in donkey's milk	[32]
**a-casein**	αs_1-casein (increased with cow milk adulteration)	Analyze mare milk adulteration with cow milk	Mare	nLC-MS/MS (+) (DIA 395 –1205 m/z, DDA 300–1800 m/z)	5% cow's milk in mare milk	[18]
	αs_1-casein	Identification of pasteurized mare milk and mare milk powder adulterated with cow milk	Mare	nLC-MS/MS (+) (300 –1,800 m/z) (DIA) (DDA)	0.1% cow's milk in mare milk	[92]
	αs_2-casein	Protein profile from local Pakistani milk breeds animals such as Nilli-Ravi buffalo, Sahiwal cow, Kajli sheep, Beetal goat and Brela camel	Goat (Beetal), Camel (Brela)	SDS-PAGE	Not determined	[98]
		Analyze mare milk adulteration with cow milk	Mare	nLC-MS/MS (+) (DIA 395 –1205 m/z, DDA 300–1800 m/z)	5% cow's milk in mare milk	[18]
		Identification of pasteurized mare milk (PMM) and mare milk powder (MMP)	Mare	nLC-MS/MS (+) (300 –1,800 m/z) (DIA) (DDA)	0.1% cow's milk in mare milk	[92]

		adulterated with cow milk				
	β-casein	Detecting cow milk adulteration using cheese whey, comparing to SDS-PAGE	Cow	Microfluidic Chip Electrophoresis	1% whey in milk	[95]
	β-casein	Detection of adulteration of cow milk in yak milk	Yak	Indirect Competitive ELISA	1% (10 μg/mL) of cow's milk in yak's milk	[99]
		Protein profile of milk from local Pakistani breeds of milch animals such as Nilli-Ravi buffalo, Sahiwal cow, Kajli sheep, Beetal goat and Brela camel	Buffalo (Nilli-Ravi), Cow (Sahiwal), Sheep (Kajli), Goat (Beetal), Camel (Brela)	SDS-PAGE	Not determined	[98]
		Analyze mare milk adulteration with cow milk	Mare	nLC-MS/MS (+) (DIA 395 –1205 m/z, DDA 300–1800 m/z)	1% cow's milk	[18]
**β-casein**	β-casein (5 P): Found in Goat and Sheep milk.	Identification of proteins from cow, goat, and sheep milks, and quantification of adulteration of goat and sheep milks with cow milk	Goat, Sheep	MALDI TOF/ TOF (+) (5000 – 30,000 m/z)	5% of cow's milk in goat and sheep milk	[16]
	β-casein	Detection of cow milk in donkey milk	Donkey	Competitive ELISA	0,5% (v/v) of cow's milk in donkey's milk	[32]
		Identification of pasteurized mare milk and mare milk powder adulterated with cow milk	Mare	nLC-MS/MS (+) (300 –1,800 m/z) (DIA) (DDA)	0.1% cow's milk in mare milk	[92]
		Detects adulteration with soybean milk in cow milk	Cow	Fluorescence Spectroscopy	0.1% soybean's milk in cow milk	[97]
	β-casein fragments (1-68), (69-209): Found in Buffalo milk.	Detect adulteration of water buffalo, goat, and ovine milks with fresh and powdered cow milk	Buffalo, Sheep, Goat	MALDI-TOF-MS (+) (5000–20000 Da)	5% cow's milk in goat's milk	[91]
	β-casein fragments (98-207): Found in Goat and Sheep milk.	Detect adulteration of water buffalo, goat, and ovine milks with fresh and powdered cow milk	Buffalo, Sheep, Goat	MALDI -TOF-MS (+) (5000–20000 Da)	5% cow's milk in goat's milk	[91]
	β-casein-A1 (5 P), β-casein-A2 (5 P): Found in Cow milk	Identification of proteins from cow, goat, and sheep milks, and quantification of adulteration of goat	Goat, Sheep	MALDI TOF/ TOF (+) (5000 – 30,000 m/z)	5% of cow's milk in goat and sheep milk	[16]

		and sheep milks with cow milk				
**γ-casein**	γ2-caseins, γ3-caseins: Found in Cow, Buffalo, Goat, and Sheep milk.	Detect adulteration of water buffalo, goat, and ovine milks with fresh and powdered cow milk	Buffalo, Sheep, Goat	MALDI-TOF-MS (+) (5000–20000 Da)	5% cow's milk in goat's milk	[91]
**κ-casein**	κ-casein	Protein profile from local Pakistani milk breeds animals such as Nilli-Ravi buffalo, Sahiwal cow, Kajli sheep, Beetal goat and Brela camel	Buffalo (Nilli-Ravi), Sheep (Kajli)	SDS-PAGE	Not determined	[98]
	κ-casein (increased with cow milk adulteration)	Analyze mare milk adulteration with cow milk	Mare	nLC-MS/MS (+) (DIA 395 –1205 m/z, DDA 300–1800 m/z)	2% cow's milk in mare milk	[18]
	κ-casein (1 Pyr, 2 P): Found in Goat and Sheep milk.	Identification of proteins from cow, goat, and sheep milks, and quantification of adulteration of goat and sheep milks with cow milk	Goat, Sheep	MALDI TOF/ TOF (+) (5000 – 30,000 m/z)	5% of cow's milk in goat and sheep milk	[16]
	κ-casein (Protein biomarker for cow milk in Both Pasteurized Horse Milk and Horse Milk Powder)	Identification of pasteurized mare milk and mare milk powder adulterated with cow milk	Mare	nLC-MS/MS (+) (300 –1,800 m/z) (DIA) (DDA)	0.1% cow's milk in mare milk	[92]
	κ-casein-A (1 Pyr, 1 P), κ-casein-B (1 Pyr, 1 P): Found in cow milk	Identification of proteins from cow, goat, and sheep milks, and quantification of adulteration of goat and sheep milks with cow milk	Goat, Sheep	MALDI TOF/ TOF (+) (5000 – 30,000 m/z)	5% of cow's milk in goat and sheep milk	[16]
	κ-casein (cow)	Real-time detection of cow milk in goat milk.	Goat	Optical immunoassay	0.04% (v/v) cow's milk in goat's milk	[140]
	κ-casein (cow) (208 kDa) (PSS 208)	Detect cow milk in solutions, differentiating from goat milk	Goat	Quartz Crystal Microbalance (QCM) Immunosensor	1 mg/L cow's milk in goat's milk	[103]
	κ-casein	Detection of adulteration of goat's milk with cow's milk	Goat	2D Electrophoresis	2% cow's milk in goat milk	[140]
***α*-Lactalbumin**	*α*-lactalbumin fraction Bc (cow)	Predict cow milk concentration	Buffalo	Capillary Electrophoresis (CE)	LOD: 1% cow's milk in buffalo milk	[104]

		fraudulently added to buffalo milk			LOQ: 3.1% cow's milk in buffalo milk	
	α-Lactalbumin (increased with cow milk adulteration)	Analyze mare milk adulteration with cow milk	Mare	nLC-MS/MS (+) (DIA 395 –1205 m/z, DDA 300–1800 m/z)	0.1% cow's milk in mare milk	[18]
	α-lactalbumin (cow)	Identification of pasteurized mare milk and mare milk powder adulterated with cow milk	Mare	nLC-MS/MS (+) (300 –1,800 m/z) (DIA) (DDA)	1% cow's milk in pasteurized mare milk<br>10% cow's milk in mare milk powder	[92]
	α-lactalbumin	Detects adulteration with soybean milk in cow milk	Cow	Fluorescence Spectroscopy	0.1% soybean's milk in cow milk	[97]
	α-Lactalbumin (cow)	Identification of proteins from cow, goat, and sheep milks, and quantification of adulteration of goat and sheep milks with cow milk	Goat, Sheep	MALDI TOF/ TOF (+) (5000 – 30,000 m/z)	5% of cow's milk in goat and sheep milk	[16]
	α-Lactalbumin (goat)	Identification of proteins from cow, goat, and sheep milks, and quantification of adulteration of goat and sheep milks with cow milk	Goat, Sheep	MALDI TOF/ TOF (+) (5000 – 30,000 m/z)	5% of cow's milk in goat and sheep milk	[16]
	α-Lactalbumin (sheep)	Identification of proteins from cow, goat, and sheep milks, and quantification of adulteration of goat and sheep milks with cow milk	Goat, Sheep	MALDI TOF/ TOF (+) (5000 – 30,000 m/z)	5% of cow's milk in goat and sheep milk	[16]
	α-lactalbumin: Found in Cow, Buffalo, and Goat milk.	Detect adulteration of water buffalo, goat, and ovine milks with fresh and powdered cow milk	Buffalo, Sheep, Goat	MALDI-TOF-MS (+) (5000–20000 Da)	5% cow's milk in goat's milk	[91]
**β-Lactoglobulin**	β-lactoglobulin (cow)	Detecting fraudulent addition of cow milk to camel milk powder	Camel	UPLC-DAD (C4) (214 nm)	5% cow's milk camel milk powder	[94]
	β-lactoglobulin (18,198.7 Da for sheep 18,158.5 Da for goat)	Distinguish sheep from goat milk and sheep colostrum at different phases and classification of colostrum and mature milk	Sheep, Goat	liquid AP-MALDI MS	10% goat's milk in sheep milk	[131]
	β-lactoglobulin (cow)	Detect and quantify cow milk adulteration in camel milk	Camel	Enzyme-Linked Immunosorbent Assay (ELISA)	1% cow milk in camel milk	[128]
	β-lactoglobulin	Protein profile of milk from local Pakistani breeds of milch animals such as Nilli-Ravi buffalo,	Buffalo (Nilli-Ravi), Cow (Sahiwal) ,	SDS-PAGE	Not determined	[98]

		Sahiwal cow, Kajli sheep, Beetal goat and Brela camel	Sheep (Kajli), Goat (Beetal)			
	β-Lactoglobulin (increased with cow milk adulteration)	Analyze mare milk adulteration with cow milk	Mare	nLC-MS/MS (+) (DIA 395 –1205 m/z, DDA 300–1800 m/z)	1% cow's milk	[18]
	β-lactoglobulin (cow) (Protein biomarker for cow milk in Horse Milk Powder)	Identification of pasteurized mare milk (PMM) and mare milk powder (MMP) adulterated with cow milk (BM)	Mare	nLC-MS/MS (+) (300 –1,800 m/z) (DIA) (DDA)	10% cow's milk in mare milk powder	[92]
	β-lactoglobulin (in both skim milk and whey samples)	Proteins related to goat milk adulteration with cow milk	Goat	nLC-MS/MS (+) (DIA 395 –1205 m/z, DDA 300–1800 m/z)	0.1% cow's milk	[93]
	β-lactoglobulin	Detects adulteration with soybean milk in cow milk	Cow	Fluorescence Spectroscopy	0.1% soybean's milk in cow milk	[97]
	β-lactoglobulin (goat)	Identification of proteins from cow, goat, and sheep milks, and quantification of adulteration of goat and sheep milks with cow milk	Goat, Sheep	MALDI TOF/ TOF (+) (5000 – 30,000 m/z)	5% of cow's milk in goat and sheep milk	[16]
	β-lactoglobulin (sheep)	Identification of proteins from cow, goat, and sheep milks, and quantification of adulteration of goat and sheep milks with cow milk	Goat, Sheep	MALDI TOF/ TOF (+) (5000 – 30,000 m/z)	5% of cow's milk in goat and sheep milk	[16]
	β-lactoglobulin: Found in Goat milk.	Detect adulteration of water buffalo, goat, and ovine milks with fresh and powdered cow milk	Buffalo, Sheep, Goat	MALDI-TOF-MS (+) (5000–20000 Da)	5% cow's milk in goat's milk	[91]
	β-lactoglobulin-A (cow) β-lactoglobulin-B (cow)	Identification of proteins from cow, goat, and sheep milks, and quantification of adulteration of goat and sheep milks with cow milk	Goat, Sheep	MALDI TOF/ TOF (+) (5000 – 30,000 m/z)	5% of cow's milk in goat and sheep milk	[16]
**Immunoglobulin G**	Immunoglobulin G (cow)	Developed for the detection of cow IgG as a biomarker of goat milk adulteration	Goat, Sheep	Cyclic voltammetry (CV) - Label-free immunosensor- Signal readout: Differential Pulse Voltammetry (DPV) Electrochemical	0.01% cow's milk with Competitive NLISA<br><br>0.1% cow's milk with Label-free immunosensor	[105]

				impedance spectroscopy (EIS) - Competitive nanozyme-linked immunosorbent assay (NLISA) - Signal readout: Colorimetry		
	Immunoglobulins (in cow)	Detect cows' milk in milk from other species	Buffalo, Sheep, Goat	Lateral Flow Immunoassay (LFIA)	0.5% cow's milk in bufallo, sheep and goat milk	[106]
	Immunoglobulins G from cow, sheep, and goat milk	Detection of milk adulteration with milk or colostrum from different animal species using IgGs from cow, sheep, goat	Cow, Sheep, Goat	Amperometric Immunosensor	0.1% cow's milk	[107]
**Lactoferrin**	Lactoferrin	Detection and quantification of lactoferrin (LF) in various species' milk	Sheep, Goat, Cow, Donkey	HPLC-DAD (C8) (205 nm)	Not determined	[108]
	Lactoferrin (LRPVAAEIYGTK corresponding to amino acid residues 93–104)	Quantifying cow lactoferrin in various dairy products, including infant formulas and whey protein concentrates.	Cow	UHPLC-ESI-QTOF-MS (+) (50–1500 m/z) (MRM)	0.3 mg/100 mg cow's lactoferrin in infant formulas	[140]
**Glycomacropeptide (GMP)**	Casein glycomacropeptide (cGMP)	Detect milk adulteration with cheese whey, using casein glycomacropeptide (cgmp) as a marker	Cow	1. HPLC-DAD (205 nm) MALDI-TOF-MS in 6780 Da, 10.8 min<br>2. Immunochromatographic strip	2.5% whey in cow milk	[110]
	Glycomacropeptide (GMP) β-lactoglobulin	Detection of adulteration of cow milk with cheese whey	Cow	SDS-PAGE	20% whey in cow milk	[136]
	Caseinomacropeptide (CMP)	Detection and confirmation of milk adulteration with cheese whey	Cow	LC-ESI-MS/MS (+) (MRM)	1 μg/mL (CMP and pseudo-CMP in milk)	[111]
**Other types of proteins**	Cow serum albumin (BSA)	Detects adulteration with soybean milk in cow milk	Cow	Fluorescence Spectroscopy	0.1% soybean's milk in cow milk	[97]
	Beta-2-Microglobulin (B2M) (increased with cow milk adulteration)	Analyze mare milk adulteration with cow milk	Mare	nLC-MS/MS(+) (DIA 395 –1205 m/z,<br>DDA 300–1800 m/z)	1% cow's milk	[18]

Glycosylation-dependent Cell Adhesion Molecule 1 (GlyCam1) (in skim milk and whey samples)	Proteins related to goat milk adulteration with cow milk	Goat, Cow	nLC-MS/MS (+) (DIA 395 –1205 m/z, DDA 300–1800 m/z)	1% cow's milk	[93]
Glycosylation-dependent Cell Adhesion Molecule 1 (GlyCam1)	Identification of pasteurized mare milk and mare milk powder adulterated with cow milk	Mare	nLC-MS/MS (+) (300 –1,800 m/z) (DIA) (DDA)	1% cow's milk in pasteurized mare milk<br>10% cow's milk in mare milk powder	[92]
Histatin (HSTN) (increased with cow milk adulteration)	Analyze mare milk adulteration with cow milk using proteomics and metabolomics	Mare	nLC-MS/MS (+) (DIA 395 –1205 m/z, DDA 300–1800 m/z)	0.1% cow's milk	[18]
Mucin 15 (in skim milk and whey samples)	Proteins related to goat milk adulteration with cow milk	Goat	nLC-MS/MS (+) (DIA 395 –1205 m/z, DDA 300–1800 m/z)	10% cow's milk	[93]
Osteopontin (OPN)	Identification of pasteurized mare milk and mare milk powder adulterated with cow milk	Mare	nLC-MS/MS (+) (300 –1,800 m/z) (DIA) (DDA)	1% cow's milk	[92]
Polymeric Immunoglobulin Receptor (PIGR) (increased with cow milk adulteration)	Analyze mare milk adulteration with cow milk using proteomics and metabolomics	Mare	nLC-MS/MS (+) (DIA 395 –1205 m/z, DDA 300–1800 m/z)	1% cow's milk	[18]
Polymeric Immunoglobulin Receptor (PIGR) (in skim milk sample)	Proteins related to goat milk adulteration with cow milk	Goat	nLC-MS/MS (+) (DIA 395 –1205 m/z, DDA 300–1800 m/z)	1% cow's milk	[93]
Proteoso peptones p.p.8.I.: Found in Cow milk	Detect adulteration of water buffalo, goat, and ovine milks with fresh and powdered cow milk	Buffalo, Sheep, Goat	MALDI-TOF-MS (+) (5000–20000 Da)	5% cow's milk in goat's milk	[91]
Secretoglobin Family 1D Member (SCGB1D)	Proteins related to goat milk adulteration with cow milk	Goat	nLC-MS/MS (+) (DIA 395 –1205 m/z, DDA 300–1800 m/z)	0.1% cow's milk	[93]

	(in skim milk and whey samples)					
	Secretoglobin Family 1D Member (SCGB1D) (increased with cow milk adulteration)	Analyze mare milk adulteration with cow milk using proteomics and metabolomics	Mare	nLC-MS/MS (+) (DIA 395 –1205 m/z, DDA 300–1800 m/z)	1% cow's milk	[18]
	Serotransferrin (TF)	Identification of pasteurized mare milk and mare milk powder adulterated with cow milk	Mare	nLC-MS/MS (+) (300 –1,800 m/z) (DIA) (DDA)	10% cow's milk	[92]
	Serpin A3-1 (in whey sample)	Proteins related to goat milk adulteration with cow milk	Goat	nLC-MS/MS (+) (DIA 395 –1205 m/z, DDA 300–1800 m/z)	10% cow's milk	[93]
	Zinc-α-2-glycoprotein (in whey sample)	Proteins related to goat milk adulteration with cow milk	Goat	nLC-MS/MS (+) (DIA 395 –1205 m/z, DDA 300–1800 m/z)	1% cow's milk	[93]
	Zinc-α-2-glycoprotein (AZGP1)	Identification of pasteurized mare milk and mare milk powder adulterated with cow milk	Mare	nLC-MS/MS (+) (300 –1,800 m/z) (DIA) (DDA)	1% cow's milk in pasteurized mare milk<br>10% cow's milk in mare milk powder	[92]
	α-2-HS-glycoprotein (in whey sample)	Novel proteins related to goat milk adulteration with cow milk	Goat, Cow	nLC-MS/MS (+) (DIA 395 –1205 m/z, DDA 300–1800 m/z)	0.1% cow's milk	[93]
	A total of 1,001 proteins were quantified in goat milk collected from **3 habitats of China:**<br>1. Guangdong (GD)<br>2. Inner Mongolia (IM)<br>3. Shannxi (SX)<br>Differentially expressed proteins for GD versus IM	Identify fat globule membrane (MFGM) proteins in Saanen goat milk samples collected from 3 different habitats in China	Goat	HPLC (SWATH)	Statistical analysis (PCA, Heatmap and Hierarchical Clustering)	[137]

	were identified to be 81 GD versus SX, were identified to be 91 IM versus SX were identified to be 44					
	Buffalo's milk protein fraction	Adulteration of cow's milk with buffalo 's milk	Buffalo	competitive Lateral Flow Immunoassay (LFIA)	5% buffalo's milk in cow milk	[138]
	Intact proteins combined with statistical analysis: **Hierarchical clustering, correlation analysis** and **PCA**	Identification of donkey's milk (DM) and goat's milk (GM) adulteration by cow's milk (CM), ewe milk (EM) and buffalo milk (BM).	Donkey, Goat	MALDI-TOF-MS (+) (2000-25,000 Da)	0.5% cow's milk in donkey's and goat's milk	[125]
	Ranges of peptide-protein spectra (500–4,000 Da; 4–20 kDa) with **linear model with lasso regularization (GLM-Lasso)** prediction model	Detecting cow milk in caprine and ovine milk from different Czech farms.	Goat, Sheep, Cow	MALDI-TOF-MS (+) (500-17,000 Da)	17.0% cow's milk in sheep milk	[123]
	m/z 1265 and 1335 (only in cow milk) with LDA-based machine-learning prediction models	Determining milk adulteration of cow milk added to goat milk	Goat, Sheep, Cow, Camel Fresh	AP-MALDI-TOF-MS (+) (100–2000 m/z)	5% cow's milk	[119]
	The relative areas of peaks of N-glycans:<br>1. A3 (1825.74 m/z) gradually <u>increased</u> with the increasing proportion of cow milk<br>2. FA2G2S	Adulteration of cow milk in goat milk	Cow, Goat	MALDI-TOF MS/MS	5% cow's milk in goat milk	[130]

	(2215.89 m/z) gradually increased with the increasing proportion of cow milk<br>3. A3G1 (2254.21 m/z) gradually decreased with the increasing proportion of cow milk<br>4. A3BG3S(3)S (2938.89 m/z) gradually decreased with the increasing proportion of cow milk					
	Two distinct datasets containing full protein and peptide fingerprints were combined to improve the discriminating ability of principle component analysis (PCA). The ***α*-lactalbumin glycosylated polypeptide** was thought to be the potential component causing the distinction.	Detect milk powder adulterated to liquid milk	Cow	1. **Intact protein analysis**: UPLC-ESI-QTOF-MS (C4) (+) (50-1,500 m/z)<br>2. **Hydrolyzed peptide analysis**: UPLC-ESI-QTOF-MS (+) (50-1,500 m/z)	0.5% milk powder in liquid whole milk	[129]
	• β-conglycinin, Glycinin: Soy<br>• Vincilin, Convicilin, Legumin: Pea	Identification of the (hydrolyzed) plant protein in adulterated milk	Cow	1. nano-HPLC-MS/MS (+) (300–1,800 m/z)<br>2. SDS-PAGE	soy protein and hydrolyzed wheat protein at the level of 0.5%–4%<br>pea protein at the level of 2%	[127]

• Glutenin, Gliadin: Wheat		hydrolyzed rice protein at the level of 4%

References

16. Tehrani, T.; Pont, L.; Benavente, F. Rapid Detection and Quantification of Milk Adulteration Using MALDI-MS Protein Profiling and Multivariate Calibration. *Journal of Food Composition and Analysis* **2024**, *130*, 106147, doi:10.1016/j.jfca.2024.106147.

18. Ji, Z.; Zhang, J.; Deng, C.; Hu, Z.; Du, Q.; Guo, T.; Wang, J.; Fan, R.; Han, R.; Yang, Y. Identification of Mare Milk Adulteration with Cow Milk by Liquid Chromatography-High Resolution Mass Spectrometry Based on Proteomics and Metabolomics Approaches. *Food Chemistry* **2023**, *405*, 134901, doi:10.1016/j.foodchem.2022.134901.

32. Pizzano, R.; Salimei, E. Isoelectric Focusing and ELISA for Detecting Adulteration of Donkey Milk with Cow Milk. *J. Agric. Food Chem.* **2014**, *62*, 5853–5858, doi:10.1021/jf5025533.

91. Sassi, M.; Arena, S.; Scaloni, A. MALDI-TOF-MS Platform for Integrated Proteomic and Peptidomic Profiling of Milk Samples Allows Rapid Detection of Food Adulterations. *J. Agric. Food Chem.* **2015**, *63*, 7093–7093, doi:10.1021/acs.jafc.5b03524.

92. Ji, Z.; Zhang, J.; Deng, C.; Guo, T.; Han, R.; Yang, Y.; Zang, C.; Chen, Y. Identification of Pasteurized Mare Milk and Powder Adulteration with Bovine Milk Using Quantitative Proteomics and Metabolomics Approaches. *Food Chemistry: X* **2024**, *22*, 101265, doi:10.1016/j.fochx.2024.101265.

93. Fan, R.; Xie, S.; Wang, S.; Yu, Z.; Sun, X.; Du, Q.; Yang, Y.; Han, R. Identification Markers of Goat Milk Adulterated with Bovine Milk Based on Proteomics and Metabolomics. *Food Chemistry: X* **2023**, *17*, 100601, doi:10.1016/j.fochx.2023.100601.

94. Li, L.; Wang, J.; Li, M.; Yang, Y.; Wang, Z.; Miao, J.; Zhao, Z.; Yang, J. Detection of the Adulteration of Camel Milk Powder with Cow Milk by Ultra-High Performance Liquid Chromatography (UPLC). *International Dairy Journal* **2021**, *121*, 105117, doi:10.1016/j.idairyj.2021.105117.

95. Santos, A.S.D.O.D.; Pereira, H.P.; Fogaça, G.N.; Meurer, V.M.; Furtado, M.A.M.; Borges, C.A.V.; Weller, M.M.D.C.A.; Martins, M.F. Separation and Quantification of Milk Proteins with the Addition of Cheese Whey by Lab-on-a-Chip. *Pesq. agropec. bras.* **2023**, *58*, e03099, doi:10.1590/s1678-3921.pab2023.v58.03099.

96. Trimboli, F.; Morittu, V.M.; Cicino, C.; Palmieri, C.; Britti, D. Rapid Capillary Electrophoresis Approach for the Quantification of Ewe Milk Adulteration with Cow Milk. *Journal of Chromatography A* **2017**, *1519*, 131–136, doi:10.1016/j.chroma.2017.08.075.

97. Zhang, L.; Hou, J.; Zhou, H.; Nawaz, M.A.H.; Li, Y.; Huang, H.; Yu, C. Identification of Milk Adulteration by a Sensor Array Based on Cationic Polymer Induced Aggregation of a Perylene Probe. *Food Chemistry* **2021**, *343*, 128492, doi:10.1016/j.foodchem.2020.128492.

98. Yasmin, I.; Iqbal, R.; Liaqat, A.; Khan, W.A.; Nadeem, M.; Iqbal, A.; Chughtai, M.F.J.; Rehman, S.J.U.; Tehseen, S.; Mehmood, T.; et al. Characterization and Comparative Evaluation of Milk Protein Variants from Pakistani Dairy Breeds. *Food Sci Anim Resour* **2020**, *40*, 689–698, doi:10.5851/kosfa.2020.e44.

99. Ren, Q.R.; Zhang, H.; Guo, H.Y.; Jiang, L.; Tian, M.; Ren, F.Z. Detection of Cow Milk Adulteration in Yak Milk by ELISA. *Journal of Dairy Science* **2014**, *97*, 6000–6006, doi:10.3168/jds.2014-8127.

103. Sakti, S.P.; Chabibah, N.; Ayu, S.P.; Padaga, M.C.; Aulanni'am, A. Development of QCM Biosensor with Specific Cow Milk Protein Antibody for Candidate Milk Adulteration Detection. *Journal of Sensors* **2016**, *2016*, 1–7, doi:10.1155/2016/1807647.

104. Trimboli, F.; Costanzo, N.; Lopreiato, V.; Ceniti, C.; Morittu, V.M.; Spina, A.; Britti, D. Detection of Buffalo Milk Adulteration with Cow Milk by Capillary Electrophoresis Analysis. *Journal of Dairy Science* **2019**, *102*, 5962–5970, doi:10.3168/jds.2018-16194.
105. Seddaoui, N.; Attaallah, R.; Amine, A. Development of an Optical Immunoassay Based on Peroxidase-Mimicking Prussian Blue Nanoparticles and a Label-Free Electrochemical Immunosensor for Accurate and Sensitive Quantification of Milk Species Adulteration. *Microchim Acta* **2022**, *189*, 209, doi:10.1007/s00604-022-05302-9.
106. Galan-Malo, P.; Mendiara, I.; Razquin, P.; Mata, L. Validation of a Rapid Lateral Flow Method for the Detection of Cows′ Milk in Water Buffalo, Sheep or Goat Milk. *Food Additives & Contaminants: Part A* **2018**, *35*, 609–614, doi:10.1080/19440049.2018.1426886.
107. Ruiz-Valdepeñas Montiel, V.; Povedano, E.; Benedé, S.; Mata, L.; Galán-Malo, P.; Gamella, M.; Reviejo, A.J.; Campuzano, S.; Pingarrón, J.M. Disposable Amperometric Immunosensor for the Detection of Adulteration in Milk through Single or Multiplexed Determination of Bovine, Ovine, or Caprine Immunoglobulins G. *Anal. Chem.* **2019**, *91*, 11266–11274, doi:10.1021/acs.analchem.9b02336.
108. Tsakali, E.; Chatzilazarou, A.; Houhoula, D.; Koulouris, S.; Tsaknis, J.; Van Impe, J. A Rapid HPLC Method for the Determination of Lactoferrin in Milk of Various Species. *Journal of Dairy Research* **2019**, *86*, 238–241, doi:10.1017/s0022029919000189.
110. Vera-Bravo, R.; Hernández, A.V.; Peña, S.; Alarcón, C.; Loaiza, A.E.; Celis, C.A. Cheese Whey Milk Adulteration Determination Using Casein Glycomacropeptide as an Indicator by HPLC. *Foods* **2022**, *11*, 3201, doi:10.3390/foods11203201.
111. Motta, T.M.C.; Hoff, R.B.; Barreto, F.; Andrade, R.B.S.; Lorenzini, D.M.; Meneghini, L.Z.; Pizzolato, T.M. Detection and Confirmation of Milk Adulteration with Cheese Whey Using Proteomic-like Sample Preparation and Liquid Chromatography–Electrospray–Tandem Mass Spectrometry Analysis. *Talanta* **2014**, *120*, 498–505, doi:10.1016/j.talanta.2013.11.093.
119. Piras, C.; Hale, O.J.; Reynolds, C.K.; Jones, A.K.; Taylor, N.; Morris, M.; Cramer, R. Speciation and Milk Adulteration Analysis by Rapid Ambient Liquid MALDI Mass Spectrometry Profiling Using Machine Learning. *Sci Rep* **2021**, *11*, 3305, doi:10.1038/s41598-021-82846-5.
123. Rysova, L.; Cejnar, P.; Hanus, O.; Legarova, V.; Havlik, J.; Nejeschlebova, H.; Nemeckova, I.; Jedelska, R.; Bozik, M. Use of MALDI-TOF MS Technology to Evaluate Adulteration of Small Ruminant Milk with Raw Bovine Milk. *Journal of Dairy Science* **2022**, *105*, 4882–4894, doi:10.3168/jds.2021-21396.
125. Di Girolamo, F.; Masotti, A.; Salvatori, G.; Scapaticci, M.; Muraca, M.; Putignani, L. A Sensitive and Effective Proteomic Approach to Identify She-Donkey's and Goat's Milk Adulterations by MALDI-TOF MS Fingerprinting. *IJMS* **2014**, *15*, 13697–13719, doi:10.3390/ijms150813697.
127. Chi, S.-X.; Liu, B.-H.; Zhang, B.; Wang, B.-R.; Zhou, J.; Li, L.; Zhang, Y.-H.; Mu, Z. Development of an ELISA Method to Determine Adulterated Cow Milk in Camel Milk. *International Dairy Journal* **2024**, *155*, 105953, doi:10.1016/j.idairyj.2024.105953.
128. Du, L.; Lu, W.; Zhang, Y.; Gao, B.; Yu, L. Detection of Milk Powder in Liquid Whole Milk Using Hydrolyzed Peptide and Intact Protein Mass Spectral Fingerprints Coupled with Data Fusion Technologies. *Food Science & Nutrition* **2020**, *8*, 1471–1479, doi:10.1002/fsn3.1430.
129. Liu, Y.; Hu, X.; Voglmeir, J.; Liu, L. N-Glycan Profiles as a Tool in Qualitative and Quantitative Analysis of Goat Milk Adulteration. *Food Chemistry* **2023**, *423*, 136116, doi:10.1016/j.foodchem.2023.136116.
130. Piras, C.; Ceniti, C.; Hartmane, E.; Costanzo, N.; Morittu, V.M.; Roncada, P.; Britti, D.; Cramer, R. Rapid Liquid AP-MALDI MS Profiling of Lipids and Proteins from Goat and Sheep Milk for Speciation and Colostrum Analysis. *Proteomes* **2020**, *8*, 20, doi:10.3390/proteomes8030020.

131. Blanchet, L.; Smolinska, A. Data Fusion in Metabolomics and Proteomics for Biomarker Discovery. In *Statistical Analysis in Proteomics*; Jung, K., Ed.; Methods in Molecular Biology; Springer New York: New York, NY, 2016; Vol. 1362, pp. 209–223 ISBN 978-1-4939-3105-7.
136. Gustavsson, F.; Buitenhuis, A.J.; Johansson, M.; Bertelsen, H.P.; Glantz, M.; Poulsen, N.A.; Lindmark Månsson, H.; Stålhammar, H.; Larsen, L.B.; Bendixen, C.; et al. Effects of Breed and Casein Genetic Variants on Protein Profile in Milk from Swedish Red, Danish Holstein, and Danish Jersey Cows. *Journal of Dairy Science* **2014**, *97*, 3866–3877, doi:10.3168/jds.2013-7312.
137. Tacoma, R.; Fields, J.; Ebenstein, D.B.; Lam, Y.-W.; Greenwood, S.L. Characterization of the Bovine Milk Proteome in Early-Lactation Holstein and Jersey Breeds of Dairy Cows. *Journal of Proteomics* **2016**, *130*, 200–210, doi:10.1016/j.jprot.2015.09.024.
138. Li, S.; Ye, A.; Singh, H. Seasonal Variations in Composition, Properties, and Heat-Induced Changes in Bovine Milk in a Seasonal Calving System. *Journal of Dairy Science* **2019**, *102*, 7747–7759, doi:10.3168/jds.2019-16685.
140. Morton, J.M.; Auldist, M.J.; Douglas, M.L.; Macmillan, K.L. Associations between Milk Protein Concentration at Various Stages of Lactation and Reproductive Performance in Dairy Cows. *Journal of Dairy Science* **2016**, *99*, 10044–10056, doi:10.3168/jds.2016-11276.
